# Tracking the North American Asian Longhorned Beetle Invasion With Genomics

**DOI:** 10.1111/eva.70036

**Published:** 2024-11-19

**Authors:** Mingming Cui, Amanda D. Roe, Brian Boyle, Melody Keena, Yunke Wu, W. Evan Braswell, Michael T. Smith, Ben Gasman, Juan Shi, Marion Javal, Geraldine Roux, Jean J. Turgeon, Richard Hamelin, Ilga Porth

**Affiliations:** ^1^ Institut de Biologie Intégrative et des Systèmes Université Laval Quebec City Quebec Canada; ^2^ Département des sciences du bois et de la forêt Université Laval Quebec City Quebec Canada; ^3^ Natural Resources Canada, Canadian Forest Service Great Lakes Forestry Centre Sault Ste. Marie Ontario Canada; ^4^ Northern Research Station, Forest Service United States Department of Agriculture Hamden Connecticut USA; ^5^ Forest Pest Methods Laboratory, Plant Protection and Quarantine Science and Technology, Animal and Plant Health Inspection Service United States Department of Agriculture Buzzards Bay Massachusetts USA; ^6^ Insect Management and Molecular Diagnostics Laboratory, Plant Protection and Quarantine Science and Technology, Animal and Plant Health Inspection Service United States Department of Agriculture Edinburg Texas USA; ^7^ Beneficial Insects Introduction Research Lab, Agricultural Research Service United States Department of Agriculture Newark Delaware USA; ^8^ Canadian Food Inspection Agency Toronto Ontario Canada; ^9^ Key Laboratory for Silviculture and Conservation of Ministry of Education Beijing Forestry University Beijing China; ^10^ Institut National de la Recherche Agronomique, UR633 Zoologie Forestière Orléans France; ^11^ CBGP, IRD, CIRAD, INRAE, Institut Agro Montpellier Université de Montpellier Montpellier France; ^12^ Laboratoire Physiologie, Ecologie et Environnement P2E Université d'Orléans Orléans France; ^13^ Department of Forest & Conservation Sciences The University of British Columbia Vancouver British Columbia Canada

**Keywords:** biosurveillance, introduction source, invasion history, invasive species, secondary spread

## Abstract

Biological invasions pose significant threats to ecological and economic stability, with invasive pests like the Asian longhorned beetle (*Anoplophora glabripennis* Motschulsky, ALB) causing substantial damage to forest ecosystems. Effective pest management relies on comprehensive knowledge of the insect's biology and invasion history. This study uses genomics to address these knowledge gaps and inform existing biosurveillance frameworks. We used 2768 genome‐wide single nucleotide polymorphisms to compare invasive *A. glabripennis* populations in North America, using genomic variation to trace their sources of invasion and spread patterns, thereby refining our understanding of this species' invasion history. We found that most North American *A. glabripennis* infestations were distinct, resulting from multiple independent introductions from the native range. Following their introduction, all invasive populations experienced a genetic bottleneck which was followed by a population expansion, with a few also showing secondary spread to satellite infestations. Our study provides a foundation for a genome‐based biosurveillance tool that can be used to clarify the origin of intercepted individuals, allowing regulatory agencies to strengthen biosecurity measures against this invasive beetle.

## 1 Introduction

Biological invasions pose a significant threat to the ecological stability of our forests (Aukema et al. 2011; Pejchar and Mooney 2009; Pyšek et al. 2020) and are considered one of the greatest threats to biodiversity (Clavero and Garcia‐Berthou 2005; Mainka and Howard 2010). Costs of biological invasions are equivalent to natural disasters (Turbelin et al. 2023) and recent invasions of the emerald ash borer (*Agrilus planipennis*) and hemlock wooly adelgid (*Adelges tsugae*) in North America have highlighted the devastating long‐term impacts of invasive insects once these pests become established (Eschtruth, Evans, and Battles 2013; Herms and McCullough 2014; Holmes et al. 2009). Therefore, we must focus on preventing invasions and rapidly responding to new invasive pests to reduce the likelihood of their establishment (Epanchin‐Niell and Liebhold 2015). However, these proactive management approaches require detailed knowledge of each pest and its invasion pathway to establish strategies that will reduce the likelihood of future introductions (Bilodeau et al. 2019; Hamelin and Roe 2020; Roe et al. 2019).

Genomics, when integrated into a robust biosurveillance framework, can fill critical knowledge gaps, support proactive management of invasions, and improve global biosecurity (Roe et al. 2019; van Rees et al. 2022). Highly abundant genomic markers, such as single nucleotide polymorphisms (SNPs) obtained through genotyping‐by‐sequencing (GBS) (Elshire et al. 2011), can provide detailed knowledge on invasive pest biology, including insights to the invasion history, pathways of introduction, and the regional sources of invasion (Hamelin and Roe 2020). For example, genome‐wide SNPs resolved invasion pathways for *Aedes aegypti* and showed that many individuals had signatures of insecticide resistance, highlighting the risk of relying solely on these products to prevent their spread (Schmidt et al. 2019). In another invasive mosquito, *Aedes albopictus*, human‐assisted transport and road corridors were identified as important pathways for spread using highly variable genomic markers (Sherpa et al. 2020). Picq et al. (2018) showed that genome‐wide markers could reliably trace the population sources for *Lymantria dispar asiatica* and *L. d. japonica* and then used these data to identify the sources of intercepted moths, providing detailed knowledge of historic invasions and a foundation for a genomic‐based assay (Picq et al. 2023). These studies, among many others, highlight the breadth of knowledge that can be gained from genomic data on the invasion process and its ability to inform management responses to these threats.

The Asian longhorned beetle (Cerambycidae: *Anoplophora glabripennis* Motschulsky) is a polyphagous wood‐boring beetle introduced to hardwood forests in North America and Europe (Blackburn et al. 2020; Javal et al. 2017; Meng, Hoover, and Keena 2015). Recent work by Cui et al. (2022) described native *A. glabripennis* population variation using genome‐wide SNPs and delineated distinct population structure among regional populations. They successfully assigned *A. glabripennis* individuals to regional groups with a limited number of SNPs, demonstrating the ability to use these genomic markers to trace individuals to known source populations.

**FIGURE 1:**
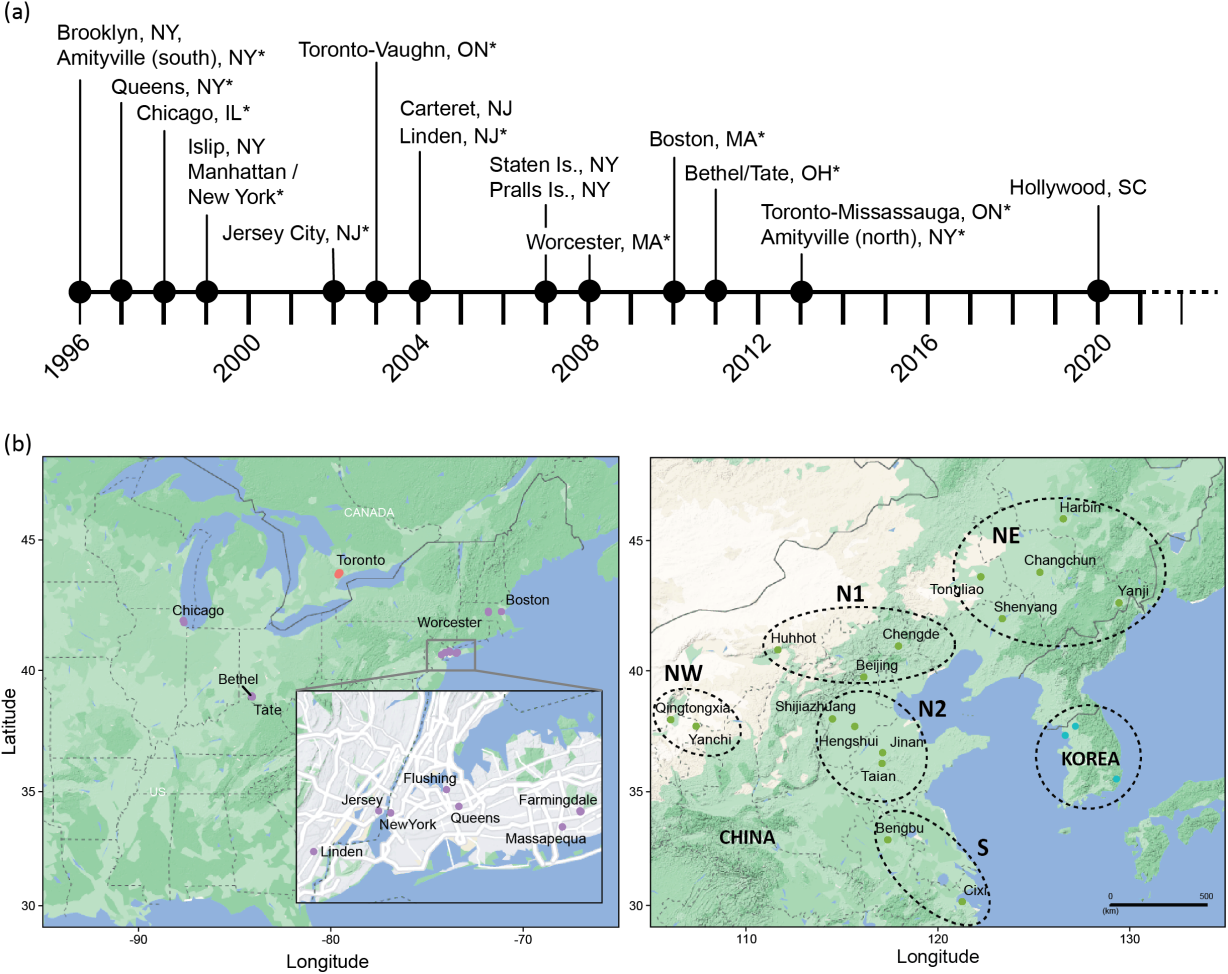
Detection history of the North American *A. glabripennis* infestations and location of sampling areas for this study. (a) Date of detection and location of *A. glabripennis* infestations in North America. Infestations with (*) were included in the genomic analysis. MA, Massachusetts; OH, Ohio; IL, Illinois; NJ, New Jersey; NY, New York; SC, South Carolina. (b) Sampling map. The left panel shows geographic sampling in the invasive range, USA (purple) and Canada (red). Toronto (includes Toronto/Vaughan and Toronto/Mississauga), Amityville (south) (includes Massapequa, NY), Amityville (north) (includes Farmingdale, NY). The right panel shows sampling in the native range: China (green) and Korea (cyan). The different regions within China are illustrated by dashed ovals: North Plain region (N1, N2), Northwest (NW), Northeast (NE), South (S), as defined in Cui et al. (2022).

Since its discovery in 1996 in Brooklyn, New York (USA), a number of *A. glabripennis* infestations have been detected in North America (Figure 1a). Knowledge of the source and pathway of introduction for each infestation can provide valuable knowledge to guide management efforts and regulation of this species. Eradication is used to manage *A. glabripennis* in North America (Smith et al. 2009; Trotter III and Hull‐Sanders 2015; Turgeon et al. 2022) and while established *A. glabripennis* populations have been successfully eradicated in several locations (Eyre and Barbrook 2021; Liebhold et al. 2016), these efforts are still ongoing in other infestations (Coyle et al. 2021). When a new infestation is detected, it is necessary to distinguish between unique invasions and satellite infestations caused by secondary spread during these control efforts. The possible source(s) of invasive *A. glabripennis* populations were previously explored using a combination of microsatellites and mitochondrial DNA by Carter, Smith, and Harrison (2010), and they suggested that separate introduction events were responsible for many North American and European populations but were unable to pinpoint the probable source populations. Similarly, Javal et al. (2019) suggested that multiple independent introductions were responsible for most European populations from the native range, and although at least one European population was founded from North America. However, confidence in determining the sources for the invasive North American populations was hampered by complex population variation, limited information in the selected genetic markers, and low sample size (Carter, Smith, and Harrison 2010; Javal et al. 2019). Genomic markers, like those used in Cui et al. (2022), are highly variable and can provide a valuable insight to the history and spread of invasive *A. glabripennis* populations, thereby informing future regulatory response plans (Bilodeau et al. 2019; Hamelin and Roe 2020; van Rees et al. 2022).

Here, we explored the *A. glabripennis* invasion in North America using genome‐wide SNPs. We used genomic markers, and the native population structure described by Cui et al. (2022) to (1) characterize population structure among North American *A. glabripennis* populations, (2) trace the sources of invasion from the native range, and (3) quantify secondary spread within North America. Collectively, these data provide a clearer picture of the invasion history of *A. glabripennis* in North America.

## 2 Methods

### 2.1 Study Organism

The native range of the *A. glabripennis* is limited to China and the Korean Peninsula. The first breeding populations discovered outside of this native range occurred in 1996 in North America (Figure 1) and 2001 in Europe (Haack et al. 1996, 1997; Poland 1998; Lingafelter and Hoebeke 2002; Krehan 2002; Javal et al. 2017). It has been recorded on > 100 hardwood tree species (Sjöman, Östberg, and Nilsson 2014) with preference for *Acer, Populus, Salix, and Ulmus* (Haack 2006; Meng, Hoover, and Keena 2015; Turgeon et al. 2021). In China, *A. glabripennis* is considered a serious forest pest and is responsible for nearly 12% of the total losses attributable to forest pests and diseases, costing an estimated $1.5 billion annually (Hu et al. 2009). As such, *A. glabripennis* is considered a high‐risk invasive beetle in both native and introduced ranges (Haack et al. 2010). The typical life cycle of *A. glabripennis* spans 1 year in most regions of China, although this is strongly dependent on environmental conditions (Lingafelter and Hoebeke 2002; Wang et al. 2023). For example, in the coldest regions in China, larvae typically require 2 years to complete their development (Lingafelter and Hoebeke 2002) and up to 3 years in cooler climates (Straw et al. 2015); while warmer locations have a synchronous univoltine life cycle with a single generation per year, similar to the native range (Schmitt 2023). The development time is relatively long compared to other cerambycid beetles (Lu et al. 2013; Bybee et al. 2004) and may influence the invasion dynamics of this species (Schmitt 2023).

### 2.2 Sampling and DNA Preparation

To determine the source(s) of the invasive populations found in Canada and the United States of America, we obtained 266 specimens over multiple years (1999–2017) (Figure 1; Table S1). We also used previously published *A. glabripennis* data (BioProject ID PRJNA824548) from China and South Korea to generate a native reference collection to define possible source populations (Cui et al. 2022).

A single leg or larval thoracic muscle was used for DNA extraction from each specimen. The tissue was surface sterilized using 95% ethanol and flash frozen in liquid nitrogen before being ground using a mixer mill (Retsch MM400, Germany) at 29 Hz for 1 min. DNA was extracted from this homogenate using the DNeasy 96 Blood & Tissue Kit (Qiagen, Hilden, Germany) with an added RNAase treatment following the manufacturer's instructions. We measured DNA quality and quantity using the NanoDrop ND‐1000 spectrophotometer (Thermo Fisher Scientific, Wilmington, DE, USA) and Qubit 2.0 Fluorometer (Thermo Fisher Scientific, Wilmington, DE, USA), respectively.

### 2.3 Genotyping and Bioinformatics Processing

GBS libraries and Ion Torrent sequencing were performed at the *plateforme d'analyses génomiques* of the *Institut de Biologie Intégrative et des Systèmes* (IBIS, Université Laval, Québec, QC, Canada). Libraries for Ion Proton GBS were prepared using the procedure described by Abed et al. (2019) with the modification to include NsiI to the double digest PstI/MspI as described in de Ronne et al. (2023). Libraries were prepared for sequencing using an Ion CHEF, Hi‐Q reagents, and P1 V3 chips (ThermoFisher Scientific, Whaltham, MA, USA), and the sequencing was performed over 300 flows on an Ion Proton instrument (ThermoFisher Scientific), following manufacturer's instructions.

We used the Fast‐GBS v1.0 pipeline to process raw sequencing reads (Torkamaneh et al. 2017; Torkamaneh, Laroche, and Belzile 2016). In this pipeline, it followed several steps to process the sequencing data. First, SABRE v1.0 was used to demultiplex single‐end, 150 bp barcoded reads (Joshi 2011) and Cutadapt v2.1 (Martin 2011) was applied to remove the GBS adapter sequences. We aligned the trimmed reads, with a minimum length of 50 bp, to the *A. glabripennis* reference genome (GCA_000390285.1) (McKenna et al. 2016) using Burrows‐Wheeler Aligner v0.7.17 (Li 2013). Samtools v1.8 (Li et al. 2009) was utilized to convert the SAM files to BAM format for indexing, and finally, PLATYPUS v0.8.1.1 was used to call variants within the pipeline, using a minimum mapping quality of 10 and maximum read length of 250 bp (Rimmer et al. 2014). SNP variants were filtered using VCFtools v0.1.16 (Danecek et al. 2011) and PLINK v2.0 (Chang et al. 2015). We applied basic filters to retain biallelic SNPs and variants with a PASS flag, retaining loci with < 50% missing data and individual samples with < 20% missing data. Furthermore, we kept loci with < 10% missing data per site, a read depth > 5, and minor allele frequency > 0.05. We then pruned SNPs in a sliding window of 50 SNPs (advanced by 5 SNPs each time) with *r* ^2^ < 0.4. Finally, we removed samples based on relatedness using the KING method (Manichaikul et al. 2010) integrated in PLINK, with a cutoff of 0.25 to remove full siblings.

### 2.4 Genetic Diversity and Population Structure

To assess genetic diversity, we calculated observed heterozygosity (*H*_o_) and expected heterozygosity (*H*_e_) for individual native and invasive populations using STACKs v1.48 (Catchen et al. 2013) and then employed the Mann–Whitney *U* nonparametric test to compare the genetic diversity between the native and invasive populations. We also measured pairwise *F*_ST_ between populations using STACKS.

We performed principal component analyses (PCA) in adegenet v2.1.2 (Jombart 2008) to characterize genetic differentiation among all samples. We applied the maximum likelihood estimation method Admixture v1.3.0 (Alexander, Novembre, and Lange 2009) to estimate individual ancestry membership. This approach employs a cross‐validation to identify the optimal *K*. We ran models with an ascending number of ancestral populations, ranging from 2 to 20, using a default fivefold cross‐validation and selected the optimal *K* based on the lowest cross‐validation error.

### 2.5 Population Assignment

We applied a discriminant analysis of principal components (DAPC) in adegenet v2.1.2 (Jombart 2008; Jombart, Devillard, and Balloux 2010) to assign invasive *A. glabripennis* samples to populations from the native range. We ran the “dapc” function on all native *A. glabripennis* samples, retaining five principal components and three discriminant functions for the discriminant analysis. Based on the DAPC results obtained from the native dataset, we used the “predict.dapc” function to predict group membership results for all invasive *A. glabripennis* samples and visualized the assignment results in a contingency table.

To further explore the evolutionary relationships among samples, we computed a maximum likelihood (ML) phylogeny using 1000 bootstrap replicates under the GTRGAMMA model in RAxML v8.2.9 (Stamatakis 2014).

### 2.6 Invasion History and Recent Gene Flow

To reconstruct the invasion history of North American *A. glabripennis*, we compared different invasion scenarios using an approximate Bayesian computation (ABC) method, implemented in DIYABC v2.1.0 (Cornuet et al. 2014). This approach simulates datasets for *a priori* invasion history models and compares these simulated datasets with the observed dataset. We followed a step‐by‐step procedure as previously described by Sherpa et al. (2019). We guided our scenario selection using results from previous genetic studies (Cui et al. 2022; Javal et al. 2019) and our current results.

The first three steps sequentially defined the divergence history of the native populations, while the next five steps identified the optimal invasion scenario for each invasive population (see Appendix S1 for further details). We only considered invasive populations with more than four individuals and a uniform genetic composition, that is, populations in which all individuals had a similar genetic composition; hence, we excluded populations from New York and New Jersey due to their demonstrated genetic complexity (see Admixture results). Based on the estimated population structure, we did not include a migration scenario in our DIYABC analyses. In the final step, we synthesized the previously inferred origins of each invasive population to construct a comprehensive evolutionary scenario. This scenario integrated all populations, and we then estimated posterior parameters, such as effective population sizes, population divergent time, and bottleneck duration. We simulated 20,000 datasets for each scenario, selecting all summary statistics for SNP loci in DIYABC. We evaluated each model (scenarios and/or associated priors) using PCA. In this process, we projected both the observed and simulated datasets onto a PCA space using the genetic summary statistics as components of the feature vector. We anticipated overlap between the observed and simulated datasets, supporting the validity of our models. For model selection and parameter estimation, we used 1000 trees simulated in DIYABC Random Forest v1.1.27 (Collin et al. 2021), an extended version of DIYABC that employs decision trees to facilitate model selection (Pudlo et al. 2016). This method generated classification votes for each scenario and allows us to estimate the posterior probability for the selected scenario.

We measured recent migration rates between populations (the proportion of individuals that are immigrants per generation) using BA3‐SNPS v1.1 (Mussmann et al. 2019), modified from BayesAss v3.04 (Wilson and Rannala 2003), which estimates recent gene flow between populations using Bayesian Markov Chain Monte Carlo resampling. We included only populations that contains more than 15 individuals, as BayesAss assumes low migration rates, which can be difficult to estimate accurately with smaller sample sizes (Meirmans 2014). First, we ran the analysis with the default value of each mixing parameter (i.e., 0.1). Based on the acceptance rates, we adjusted the mixing parameters until acceptance rates fell within the suggested range of ~0.2–0.6 (Wilson and Rannala 2003). We then performed 10 longer, independent runs with adjusted mixing parameters for allele frequencies of 0.5 and inbreeding coefficient of 0.01. Each run consisted of using 20 million iterations, with a burn‐in of five million, sampled every 1000 iterations, and produced trace files with the ‐t flag. We calculated the Bayesian deviance as suggested by Meirmans (2014) to select the best run. Run #5 (Figure S1) had the lowest deviance value and was selected for downstream parameter estimation of migration rates between *A. glabripennis* populations.

## 3 Results

### 3.1 Sequencing and Genotyping

We generated ~335 million raw reads for all invasive *A. glabripennis* samples using GBS with a triple digest library. In sum, we obtained 969,515 SNP variants through the Fast‐GBS pipeline. After variant filtering and quality control, 2768 SNPs and 490 individuals were retained. Our dataset included 156 invasive samples from North American infestations, which we combined with 331 previously published reference individuals from 16 sites in China and three putative native samples from Korea (Cui et al. 2022). A detailed summary of the number of individuals and SNPs retained after each filtering step is shown in Table S2.

### 3.2 Genetic Diversity and Population Structure

**FIGURE 2:**
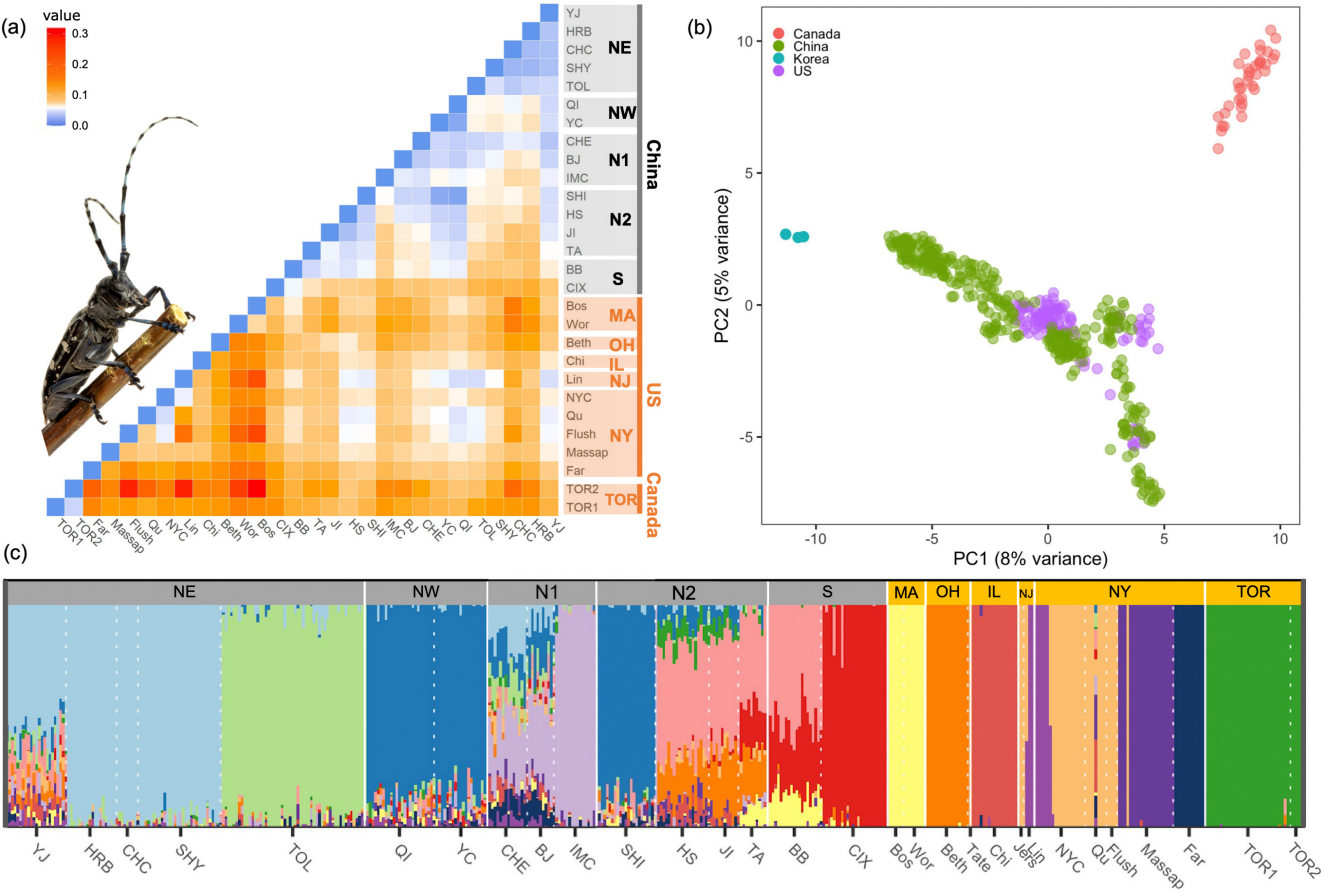
Population structure of native and North American invasive *A. glabripennis*. (a) Pairwise *F*_ST_ between populations (populations with < 4 individuals not shown), with a color ramp indicating degree of differentiation (blue = low, red = high) (see Table S4 for *F*_ST_ values). The native range includes YJ, Yanji; HRB, Harbin; CHC, Changchun; SHY, Shenyang; TOL, Tongliao; QI, Qingtongxia; YC, Yanchi; CHE, Chengde; BJ, Beijing; IMC, Huhhot; SHI, Shijiangzhuang; HS, Hengshui; JI, Jinan; TA, Taian; BB, Bengbu; CIX, Cixi. Invasive range includes Bos, Boston; Wor, Worcester; Beth, Bethel; Chi, Chicago; Lin, Linden; NYC, New York; Qu, Queen; Flush, Flushing; Massap, Massapequa; Far, Farmingdale; TOR, Toronto. See Figure 1 for locations. (b) Principal component analysis of all *A. glabripennis* populations, color‐coded by country. (c) Admixture bar plots showing the proportion of genetic membership ancestry for each individual, represented as vertical bars colored according to their estimated ancestry within each cluster. Optimal clustering is at *K* = 14 (see Figure S6 for *K* = 3 to *K* = 14). Image of an *A. glabripennis* adult was provided by Dr. Brent Sinclair.

We found that genetic diversity was generally lower in the invasive populations compared to those in the native range. *H*_o_ ranged between 0.259 and 0.326 for native populations and 0.151 and 0.270 for invasive North American populations; *H*_e_ ranges between 0.217 and 0.295 for the native populations and 0.132 and 0.235 for the invasive populations (Table 1; Table S3). The Mann–Whitney *U*‐test confirmed such differences for both *H*_o_ and *H*_e_ estimates. Specifically, the *H*_o_ in native populations (mean = 0.291) was significantly higher (*p* = 8.58e^−07^) compared to invasive populations (mean = 0.223). Similarly, *H*_e_ in native populations (mean = 0.260) was also significantly higher than in invasive populations (mean = 0.184, *p* = 2.51e^−06^). Genetic distance between populations was represented by pairwise *F*_ST_ values (Figure 2a). The average *F*_ST_ between the invasive populations was 0.1547 ± 0.0075, while between native reference populations, it was lower (0.0598 ± 0.0021), and between native and invasive populations, it was 0.096 ± 0.0021 (Table S4).

**TABLE 1:** Sampling location and genetic diversity of *A. glabripennis* infestations in North America.

Country	State/Province	Population	ID	*N*	Year sampled	*H*_o_	*H*_e_
USA	IL	Chicago	Chi	18	1999	0.254 ± 0.005	0.235 ± 0.004
	MA	Boston	Bos	6	2010	0.204 ± 0.006	0.153 ± 0.004
		Worcester	Wor	5	2008	0.234 ± 0.006	0.183 ± 0.004
		Worcester	Wor	3	2009	—	—
	NJ	Jersey City	Jers	2	2003	—	—
		Linden	Lin	4	2006	0.225 ± 0.006	0.165 ± 0.004
	NY	Farmingdale	Far	12	2013	0.270 ± 0.006	0.218 ± 0.004
		Flushing	Flush	4	2000	0.211 ± 0.006	0.163 ± 0.004
		Massapequa	Massap	14	2005	0.254 ± 0.005	0.235 ± 0.004
		Massapequa	Massap	1	2006	—	—
		Massapequa	Massap	4	2007	0.236 ± 0.006	0.184 ± 0.004
		NYC	NYC	7	2003	0.205 ± 0.005	0.183 ± 0.004
		NYC	NYC	1	2004	—	—
		NYC	NYC	1	2005	—	—
		NYC	NYC	4	2008	0.227 ± 0.006	0.170 ± 0.004
		NYC	NYC	5	2009	0.157 ± 0.006	0.120 ± 0.004
		Queens	Qu	3	1999	—	—
		Queens	Qu	4	2000	0.206 ± 0.006	0.165 ± 0.004
		Queens	Qu	8	2001	—	—
	OH	Bethel	Beth	5	2011	0.257 ± 0.006	0.214 ± 0.004
		Bethel	Beth	4	2012	0.244 ± 0.006	0.200 ± 0.004
		Bethel	Beth	2	2013	—	—
		Bethel	Beth	3	2014	—	—
		Bethel	Beth	16	2015	—	—
		Tate	Tate	1	2017	—	—
Canada	ON	Toronto/Vaughan	TOR1	32	2004	0.234 ± 0.005	0.220 ± 0.004
		Toronto/Mississauga	TOR2	5	2013	0.151 ± 0.005	0.132 ± 0.004

*Note:* Standard errors are presented for each value. Values for populations with a sampling size fewer than four were not calculated. Indices for native populations are provided in Table S3.

Abbreviations: *H*_e_, expected heterozygosity; *H*_o_, observed heterozygosity; *N*, number of individuals.

The native *A. glabripennis* reference collection samples were divided into distinct regional clusters previously delimited by Cui et al. (2022) (Figure S2; Figure 1b): (1) Northeast region (NE), bordered by the Greater Khingan Range to the west; (2) Northwest region (NW), bordered by the Helan Mountains to the east; (3) North Plain (divided into two regions, N1 & N2); (4) the South (S), bordered by the Huai River basin in the north; and (5) South Korea. They also showed that although Shijiazhuang (SHI) is geographically located in the North Plain, it is genetically more similar to the NW region, with SHI grouping with populations QI and YC rather than with geographically proximate populations in the North Plain. When we included the invasive samples (Figure 2b; Figure S3), we observed that no North American samples were associated with native Korean populations, USA samples formed groups nested within regions in China, and Toronto samples formed a single distinct cluster (Figures S3–S5).

In our admixture analysis, we selected *K* = 14 as the optimal value for the combined dataset of native and invasive populations (Figure S6, showing *K* values from 3 to 14). Both our admixture results (Figure 2c) and PCA clustering (Figure S5) were largely congruent for the invasive populations, with a few exceptions. Three of our invasive populations (TOR, OH, IL) formed distinct and uniform admixture plots and PCA clusters across sites and sampling years (Figure 2c; Figure S5). The MA population showed a distinct and uniform admixture plot; however, we observed temporal variation among individuals in the PCA plots (Figure S5c). For example, all individuals collected from Worcester, MA, in 2008 formed a tight group nested within the South cluster, along with two individuals from Boston, MA, in 2010. The remaining samples (Worcester 2009 and Boston 2010) formed a loose group with no clear source (Figure S5c).

We also observed greater genetic complexity in our NY and NJ samples compared to the other invasive populations. The NY infestation was the oldest and most extensive, with samples spanning 14 years (1999–2013), and we observed spatial and temporal genetic variation among collection locations and time points. For example, the early samples from New York City (1999–2009) were admixed and formed a large mixed cluster in the PCA (Figure 2c; Figure S5b). Farmingdale (2013), however, formed a distinct group, separate from the remaining New York samples in both the admixture plot and PCA. Massapequa (2005–2007), Queens (1999–2001), Flushing (2004) and New Jersey (2003, 2006) showed variable levels of admixture and clustering within the PCA analyses (Figure 2c; Figure S5b,e).

### 3.3 Population Assignment

**FIGURE 3:**
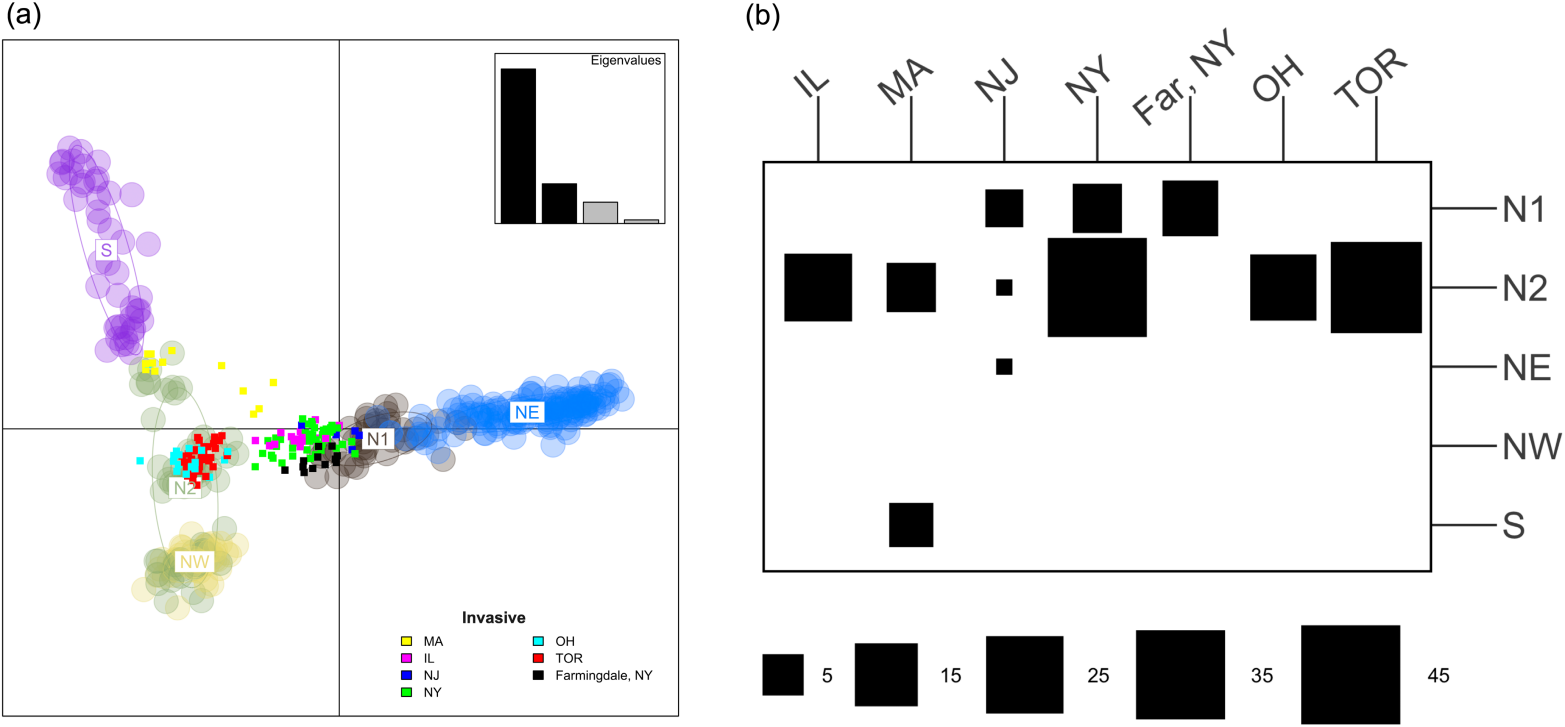
Population assignment of invasive *A. glabripennis* individuals. (a) Scatterplot of two discriminant functions showing the clustering of invasive individuals (solid squares) with reference native populations (light circles). (b) Contingency table of individual assignments to *a priori* reference native populations based on DAPC discriminant functions. Square size indicates the number of invasive individuals (columns) assigned to each native population cluster (rows). MA, Massachusetts; OH, Ohio; IL, Illinois; NJ, New Jersey; NY, New York; TOR, Toronto; N1, North Plain region one; N2, North Plain region two; NW, Northwest; NE, Northeast; S, South. Single individuals from the NJ infestation were assigned to N2 and NE. Farmingdale (Far, NY) was treated separately from other NY samples based on the admixture results.

To further refine our population assignments, we assigned invasive individuals to native reference population clusters using DAPC discriminant functions derived from a reference DAPC model (Figure 3a; Figure S7). We summarized these individual assignments in a contingency table (Figure 3b), with assignment results for each individual and their posterior membership probabilities shown in Table S5. Most invasive individuals were assigned to the N2 region in China, including all individuals in TOR, OH, and IL, as well as some individuals from MA, NJ, and NY. The remaining NY individuals were assigned to N1, including all individuals from Farmingdale, NY. A similar pattern was observed in NJ, with individuals assigned to both N1 and N2 clusters, but with the addition of one individual assigned to the NE cluster—the only North American individual associated with this region. The MA infestation also had individuals assigned to N2, with six individuals assigned to S. This is consistent with the PCA results reported earlier (Figure S5c). Notably, no invasive individuals were assigned to the NW cluster.

**FIGURE 4:**
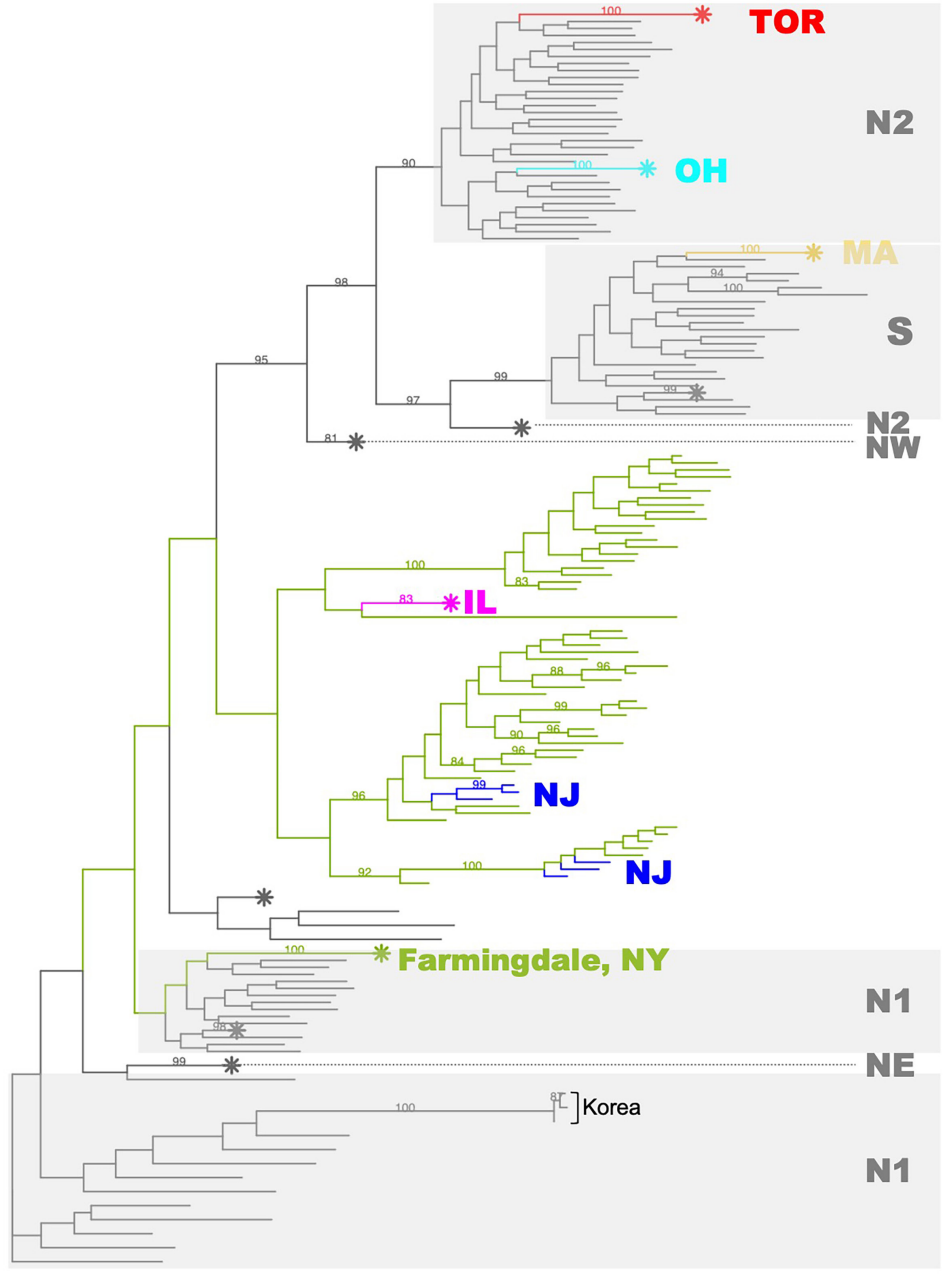
Maximum likelihood phylogenetic tree (unrooted) of the complete *A. glabripennis* dataset (*n* = 2768 SNPs). MA, Massachusetts; OH, Ohio; IL, Illinois; NJ, New Jersey; NY, New York; TOR, Toronto; N1, North Plain region one; N2, North Plain region two; NW, Northwest; NE, Northeast; S, South. Native lineages are colored in gray, while invasive populations are colored as in Figure 3. Branches marked with a “*” are collapsed clades containing multiple individuals. Branches with bootstrap values > 80% are labeled above the nodes.

To further clarify relationships between invasive individuals and native populations, we constructed an ML tree using our SNP dataset (2768 SNPs) (Figure 4). A few invasive populations were nested within native lineages with high support. Individuals from TOR and OH were well supported within the N2 lineage and MA individuals were nested within the S clade. Individuals from Farmingdale were nested within the N1 clade. The remaining individuals from NY, IL, and NJ formed a single clade with low support values, and there was no clear, well‐supported relationship to any native lineage.

### 3.4 Invasion History and Recent Gene Flow

**FIGURE 5:**
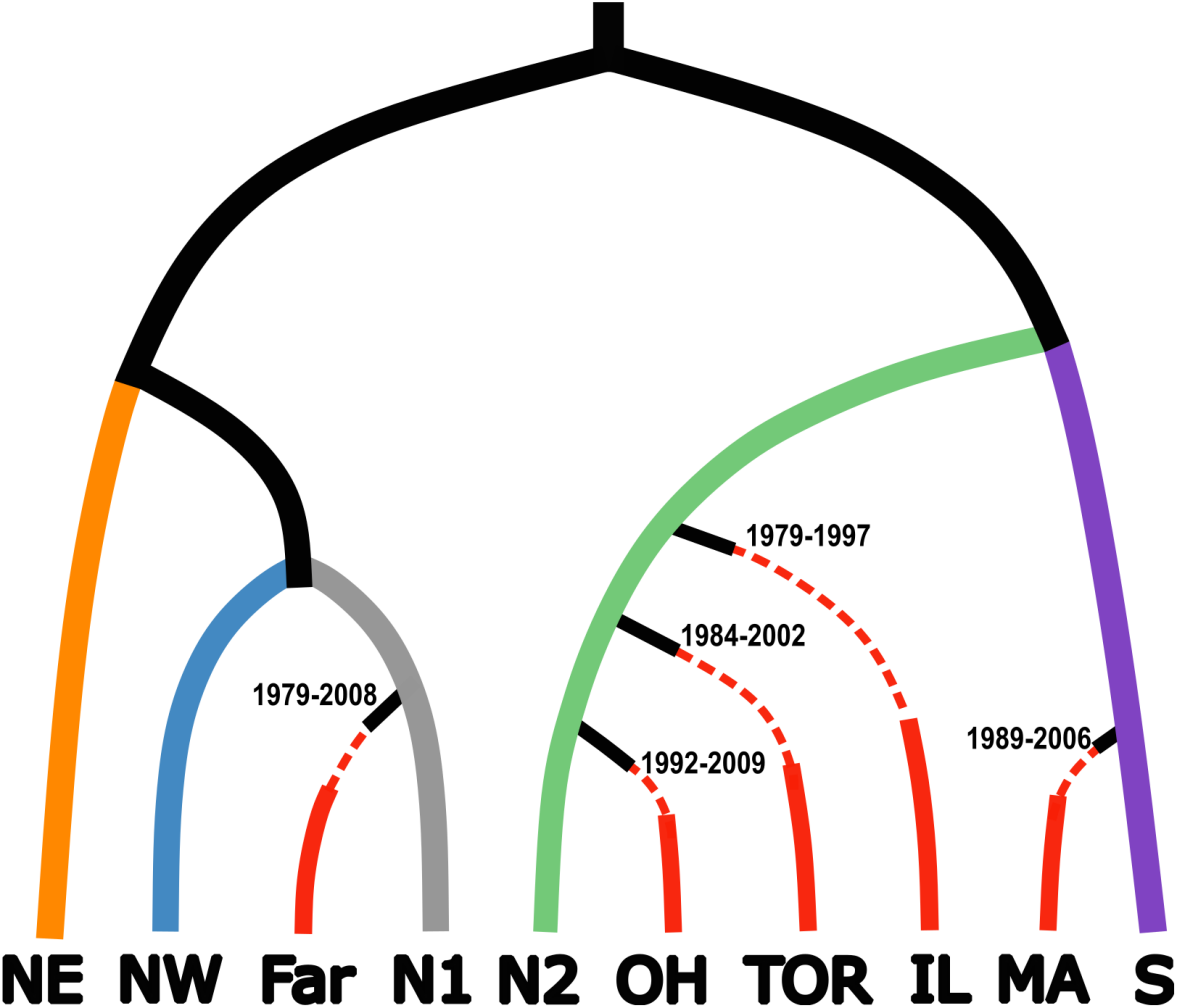
Invasion history of North American *A. glabripennis* populations. MA, Massachusetts; OH, Ohio; Far, Farmingdale, New York; TOR: Toronto; N1, North Plain region one; N2, North Plain region two; NW, Northwest; NE, Northeast; S, South. Each native region is colored individually, while all invasive populations are in red. Invasive populations experienced a bottleneck event (dashed line) followed by population expansion (solid line). The unsampled ancestry population is shown in black. Confidence intervals (95% CI, in exact years) are indicated at the point of introduction date for each invasive population (time not to scale).

We used DIYABC to reconstruct the invasion history and population demographics of five North American populations (MA, OH, IL, TOR, Far). Using an eight‐step approach to DIYABC model selection, we selected a single optimal population history model for each population (Figure S8) and a combined native + North American invasion scenario (Figure 5). The observed dataset overlapped with the simulated datasets in the PCA (Figure S9). We selected scenarios in each step with the highest classification votes. Based on this optimal model, the average introduction time of invasive populations ranged from 14 (OH) to 25 (Farmingdale) years prior to the time of sample collection (i.e., the contemporary time in the DIYABC analysis), and the estimated year of introduction for these invasive populations span from 1979 (Farmingdale, NY; IL) to 2009 (OH) (95% confidence interval) (Figure 5; Table S6). The mean predicted founding population size for the invasive populations ranged from 53 (IL) to 55 individuals (TOR) but could have started from as few as 13 individuals (OH) (Table S6). Bottleneck events lasted between 10 and 19 years (mean bottleneck duration) but could have lasted as short as 2 years (IL, TOR, MA and OH) or as long as 36 years (Farmingdale). Between the time of introduction and the sampling date, the invasive populations expanded as they grew and spread, with an average effective population size ranging from 494 (Farmingdale) to 519 (IL).

**FIGURE 6:**
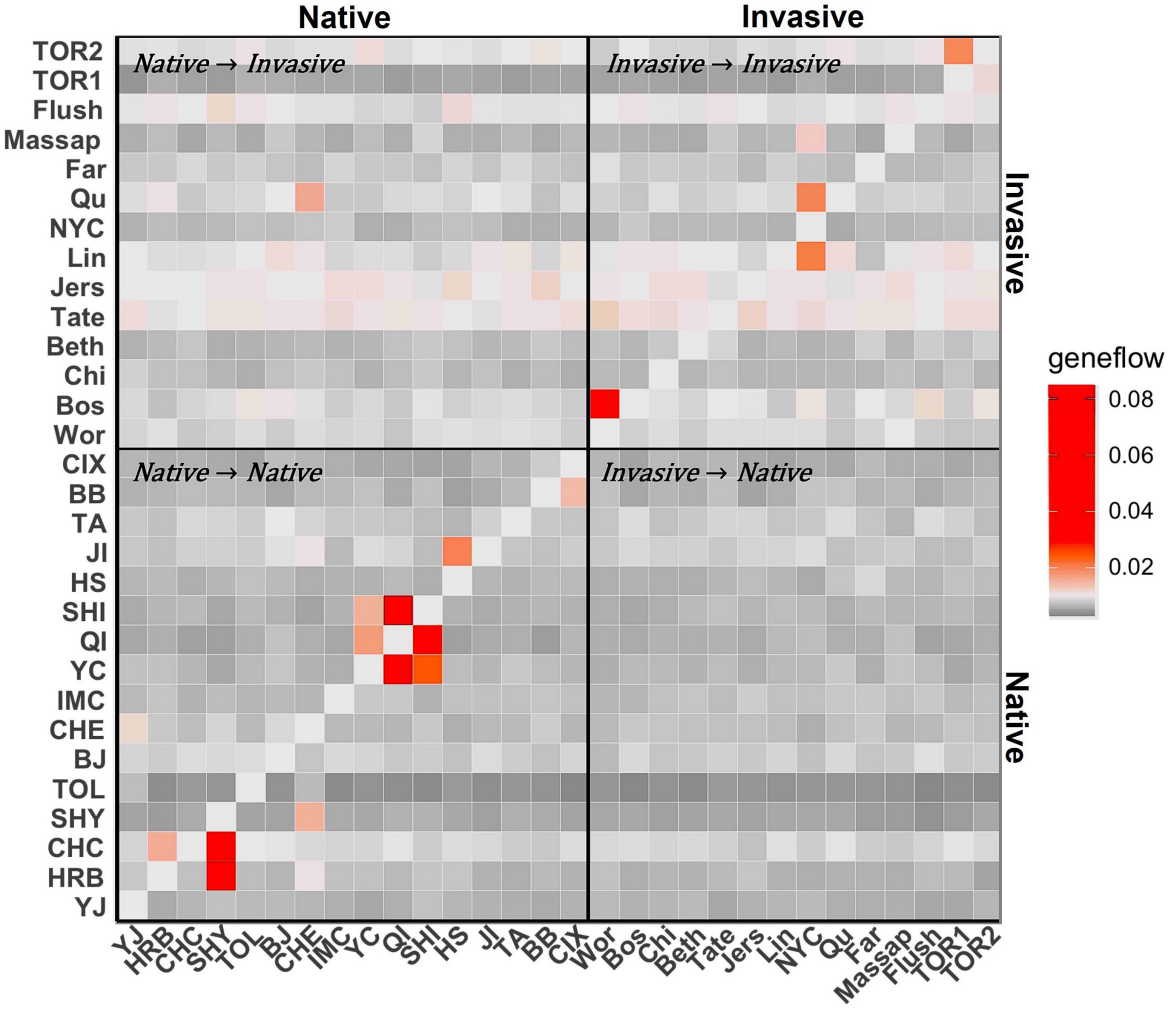
Gene flow between and within *A. glabripennis* collection sites. See Figures 1 and 2 for the locations and full names of the populations. The direction of migration is read from populations on the horizontal axis to populations on the vertical axis. The color gradient indicates migration rates, from little to no migration (gray) to increasing levels of migration (red).

We also measured migration rates between populations in both the native and invasive ranges, calculating the proportion of individuals in a population that are immigrants from other populations per generation. This reflects recent migration or colonization events. We selected Run five (Figure S1) based on its Bayesian deviance to generate point estimates and calculated the migration rate within and among native and invasive populations (Figure 6). We observed evidence of gene flow from the native range to the invaded range, with an observed migration rate of 0.0161 (proportion of individuals) from Chengde (CHE, N1) to Queens (Qu, New York), while all other rates were < 0.0123. Within the invasive range, we observed more frequent gene flow between populations, including Worcester to Boston (0.0339), New York City to Linden (0.0206), and from the first infestation of Toronto to the second (0.0196). We saw little to no contemporary gene flow from the invasive range back to the native range or among the major infestations in North America.

## 4 Discussion

The Asian longhorned beetle is an invasive insect that poses a significant threat to hardwood forests throughout its invasive range. Genome‐wide markers obtained from reduced representation libraries provide insight to the population variation among invasive *A. glabripennis* populations in North America. We showed that North American populations were structured, with multiple independent introductions from four sources in the native range. We also showed that secondary spread from initial introductions created new satellite infestations, which were likely human assisted given the relatively poor dispersal abilities of *A. glabripennis*. Finally, during their invasion, these populations experienced genetic bottlenecks followed by population expansion, demonstrating their resilience to founder effects. Collectively, our data fill important knowledge gaps about the invasion history of *A. glabripennis* in North America and can help inform future biosurveillance approaches and eradication efforts for other active infestations and in the event new breeding populations are discovered.

### 4.1 Genetic Structure and Bottlenecks in the North American *A. glabripennis* Invasion

High genetic diversity typically provides populations with the evolutionary potential to adapt to new environments. However, introduced *A. glabripennis* populations in North America (Carter, Smith, Turgeon, et al. 2009; Javal et al. 2019) and in Europe (Javal et al. 2019) showed reduced genetic diversity relative to the native populations. This phenomenon was confirmed in our results (Table 1; Table S3), and based on these findings, we included genetic bottlenecks during the initial founding within our demographic analyses (Figure 5). In fact, our demographic modeling predicted that the invasive populations were founded by relatively few individuals (Table S6). Despite this loss of diversity and limited number of founders, *A. glabripennis* populations established and expanded successfully following their introductions, as assumed in our DIYABC modeling and observed in various control efforts (e.g., Turgeon et al. 2022).

This phenomenon reflects a well‐explained “genetic paradox” where invasive species are highly successful despite their initially low genetic diversity due to bottleneck effects (Estoup et al. 2016; Schrieber and Lachmuth 2017). One explanation for this paradox is the occurrence of multiple introductions, which can restore the reduced genetic diversity during the initial invasion by mitigating inbreeding and reintroducing genetic variation (Kolbe et al. 2004). This process is common among invasive insects (Garnas et al. 2016), with recent examples in termites (Blumenfeld et al. 2021; Eyer et al. 2021), *Spodoptera frugiperda* (Tay et al. 2022), *Trichocorixa verticalis* (Ortego et al. 2021), the invasion of *Lycorma delicatula* in South Korea (Kim et al. 2021), *Roeseliana roeselii* (Kaňuch et al. 2022) and *Bemisia argentifolii* (Wongnikong, Hereward, and Walter 2021).

Interestingly, however, the restoration of genetic diversity through admixture is not a prerequisite for invasion success, as demonstrated by our findings, where most of the invasive populations appeared to have resulted from independent introduction events and thus remain genetically distinct. Similar to our study, successful invasions can occur from independent introductions, such as for the invasive Hawaiian crickets *Teleogryllus oceanicus* (Zhang et al. 2021), small hive beetles *Aethina tumida* (Liu et al. 2021), and the melon fly *Bactrocera cucurbitae* (Dupuis et al. 2018). These invasive populations may possess preadapted traits that are favorable in the invaded region, contributing to their invasion success (Estoup et al. 2016). In our study, invasive *A. glabripennis* may have benefited from the climate and host plants that are similar to those in its native range. Additionally, introduced species can undergo rapid evolutionary change (Whitney and Gabler 2008), particularly insects, which are further favored by their short generation times (Loxdale 2010; McCulloch and Waters 2023). For instance, the invasive Asian honeybee *Apis cerana* population in Australia was founded by only one colony despite a severe bottleneck, and signatures of positive selection were detected on standing genetic variation during the first few years after its invasion (Dogantzis et al. 2024). Therefore, preadaptation and/or rapid spread of advantageous alleles in the invasive populations may have contributed to the invasion success of North American *A. glabripennis*. Future studies could benefit from a more targeted approach, such as scanning for genomic signatures of adaptation on a genome‐wide scale. The presence of multiple independent introductions offers a unique opportunity to study parallel adaptation, which could provide valuable insights to the pattern, mechanism, and rate of evolutionary change in these populations.

### 4.2 Invasion Source

Successful identification of an invasion source is dependent on levels of population structure in the native range and the ability for markers to resolve this structure (Roe et al. 2019; Hamelin and Roe 2020). We detected similar invasion patterns in our North American populations as observed in previously studied European infestations (Javal et al. 2019): the European infestations were predicted to have arisen from multiple, independent introductions from the native area, and Northern populations in China are the most probable sources. However, earlier approaches were unable to achieve adequate resolution due to marker variability and low sample size (Carter, Smith, and Harrison 2009, 2010; Javal et al. 2019). Cui et al. (2022) previously delineated six native *A. glabripennis* populations within China and Korea using genome‐wide SNP markers, which we used as source populations to reconstruct the invasion history of North American populations. Using the same genome‐wide approach, we showed that four native *A. glabripennis* populations acted as potential sources for the North American invasion (N1, N2, NE, S; Figures 3, 4, 5). These results are of much higher resolution than those reported in Javal et al. (2019).

Genome‐wide markers allowed us to characterize subtle differences between invasive *A. glabripennis* populations. The genetic distances between invasive populations were more pronounced than between native populations or between invasive populations and their native sources (Figure 2). These differences may arise from stochasticity during the colonization process, including random differences in initial allele frequency, number of founders, or subsequent genetic drift (Dlugosch et al. 2015; Dlugosch and Parker 2008). A similar pattern was observed by Ciosi et al. (2008) in the invasive beetle *Diabrotica virgifera* where invasive populations showed marked genetic differences between invasive populations, even though they originated from the same source region. This variability provides an opportunity to develop biosurveillance markers that will allow us to track secondary movement and spread in *A. glabripennis*, as well as detect potential bridgehead events in *A. glabripennis* invasions (Lombaert et al. 2010).

Tracing the source of an invasion provides useful knowledge on the history and the frequency of introductions from specific regions (i.e., propagule pressure) (Simberloff 2009). To understand the observed pattern of invasion, it is important to contextualize these results with the history and population dynamics of *A. glabripennis* within the native range. Since the 1980s, *A. glabripennis* populations have rapidly expanded in China (Yan 1985). This population growth was linked with afforestation efforts in northern China as part of the Three North Shelterbelt Forest program (TNRSF) (Luo, Wen, and Xu 2003; Zhang et al. 2016). Outbreaks of *A. glabripennis* then expanded beyond the boundaries of the TNRSF project into southern regions, and *A. glabripennis* is now considered a widespread pest throughout temperate China (Haack et al. 2010). We hypothesize that the increased prevalence of *A. glabripennis* in the native range contributed to higher invasion risk and more likely invasion outcomes. Our sampling in the native range included sites within (NW, N1, NE) and outside (N2, S) the TNRSF. The earliest *A. glabripennis* detections in NY and NJ were traced to the N1, N2, and NE region, which includes both TNRSF and non‐TNRSF locations. Later infestations, such as those associated with TOR, OH, and MA, were entirely from sources outside of the TNRSF region. The N2 source region has experienced severe population outbreaks since the early 2000s (Huang et al. 2021), and this population growth in China coincides with the timing of some later invasions in North America (Figure 1). High native population density of a potential invasive increases propagule pressure and increases the probability for successful establishment of an invasive population (Lockwood, Cassey, and Blackburn 2009; Simberloff 2009). The spatiotemporal timing of native outbreaks tracks shifts in source populations for the North American incursions of *A. glabripennis* and a detailed spatiotemporal analysis of the global invasion history will provide greater insight to this hypothesis.

Human‐assisted dispersal is key to the global movement of many invasive insects, including *A. glabripennis* (Haack 2006; Ladin et al. 2023; Short et al. 2019). Larvae are found within the heartwood and can be readily transported in solid wood packing material, which includes pallets, dunnage, and spools (Greenwood et al. 2023). Port inspections continue to discover infested packaging material (Krishnankutty et al. 2020; Wu et al. 2017), despite phytosanitary measures established to curb the spread of wood‐boring invasives (Aukema et al. 2010). Our results, combined with increasing global connectivity and continued interceptions (Garnas et al. 2016; Roques et al. 2016; Wu et al. 2017), show that the risk of future *A. glabripennis* introductions remains high. Our data identified a high‐risk source region (N2: Shandong and Hebei provinces) and could help guide targeted inspections and surveillance protocols to mitigate the risk of future *A. glabripennis* introductions.

### 4.3 Secondary Spread of *A. glabripennis*

Following introduction and establishment, expansion of an invasive species is driven by secondary spread into neighboring habitats (Blackburn et al. 2011). Limiting secondary spread is often a critical component of invasive species control and management (Garnas et al. 2016; Pyšek and Richardson 2010). Adult *A. glabripennis* are considered relatively stationary and rarely disperse beyond natal or nearby hosts when conditions are favorable (Zhou, Zhang, and Lu 1984), with 98% of the individuals recaptured within 920 m of the release site (Smith et al. 2004). Unlike many invasives, eradication of *A. glabripennis* is possible and due primarily to this limited dispersal capacity (Smith et al. 2001, 2004; Turgeon et al. 2022) and relatively low reproductive rate in the invasive area (Keena 2002; but see Coyle et al. 2021). Human‐assisted movement, together with occasional natural dispersal over longer distances (Hull‐Sanders et al. 2017; Lopez et al. 2017; Javal et al. 2018), can undermine these eradication activities and cause pests to breach regulated quarantine zones (Hulme 2009; Rassati et al. 2018). For instance, Brooklyn is considered the original infestation in the New York region where it was first detected in 1996 (Sawyer 2007). Central Long Island includes several infestations which were predicted to be a result of secondary spread from the original Brooklyn population (Haack et al. 1997). Our genomic results support this hypothesis, with evidence of gene flow from NYC to the Long Island area (i.e., Massapequa, Figure 6).

Distinguishing between new introductions and satellite populations founded by secondary spread helps regulatory agencies evaluate the success of eradication efforts, establish appropriate quarantine zones, and identify high risk pathways of movement (Garnas et al. 2016). For example, we showed contemporary gene flow from Worcester to Boston (Figure 6), suggesting that Boston was a satellite population founded from secondary spread out of the Worcester infestation. Worcester is a large infestation (Dodds and Orwig 2011; Meng, Hoover, and Keena 2015) and Boston is beyond the dispersal range previously recorded in *A. glabripennis* (Smith et al. 2001, 2004); thus, it is reasonable to assume that the spread was due to human activity. Similarly, the Toronto/Mississauga infestation in 2013 (TOR2) was thought to be a satellite of the population discovered in 2003 (Turgeon et al. 2015). Our results confirmed this hypothesis, as we observed migration from Toronto/Vaughn (TOR1) into TOR2 as well as lower genetic diversity in TOR2, which would be consistent with a genetic bottleneck resulting from eradication efforts and/or founder effects. Our genomic data show that distant satellite populations can arise well beyond the dispersal range of *A. glabripennis* (< 3 km, Smith et al. 2004), and beyond the typical regulated area of an infestation (e.g., Fournier and Turgeon 2017). Knowledge of invasion pathways helps reduce uncertainty of spread dynamics and can assist with the deployment of limited surveillance resources (Melbourne and Hastings 2009; Yemshanov et al. 2017). Our data on the nature of satellite populations and secondary spread provides further support for the scenario‐based surveillance approach, which includes surveys well beyond the boundaries of the quarantine zone to capture distant, low probability spread events (Yemshanov et al. 2017).

Even with secondary spread within the North American invasion, there is limited evidence of admixture among the invasive populations (Figure 2). Admixture can increase genetic diversity and create new allele combinations that could affect the evolutionary trajectory of an invasive population (Dlugosch and Parker 2008; Rius and Darling 2014). Admixture is predicated on contact between divergent lineages and although we did not detect admixture in the majority of our populations, we observed such patterns in both the NY and NJ populations (Figure 2) which require further exploration. Given the distinct genomic signatures of each invasive population, secondary spread between infestations could potentially increase admixture within the invasive range and alter the evolutionary potential and inherent risk posed by these infestations. Admixed invasive populations have been identified as important drivers of global invasions and are frequently detected in bridgehead invasion scenarios (Lombaert et al. 2010). Bridgehead events (*sensu* Lombaert et al. 2010) occur when a successful invasive lineage acts as a source for new invasive populations, a phenomenon frequently reported in other invasive groups (Bertelsmeier 2021; Blumenfeld et al. 2021; Garnas et al. 2016; Kim et al. 2021; Ortego et al. 2021; Rius and Darling 2014). In *A. glabripennis*, a bridgehead scenario from invasive USA populations was predicted for Gien, France (Javal et al. 2019), although we cannot yet assess whether a similar situation happened in North America. A global survey of invasive *A. glabripennis* populations is needed to clarify whether additional bridgehead invasion scenarios have occurred and the nature of admixture in these two invaded regions. If *A. glabripennis* infestations are not eradicated and are allowed to persist, the risk of admixture increases, particularly if secondary spread and gene flow occurs between populations with distinct sources and unique genetic diversity. Our genomic data provide a historic baseline which can be used to assess whether genetic structure, diversity, and admixture in invasive *A. glabripennis* populations change over time.

## 5 Conclusion

The Asian longhorned beetle is a high‐risk invasive species that continues to threaten temperate forests in North America, Europe, and other parts of Asia. Over 30 global incursions of *A. glabripennis* have occurred (Haack et al. 2010), with recent invasions discovered in South Carolina and Japan (Akita et al. 2021; Coyle et al. 2021). While eradication is possible, active infestations are still being controlled and live *A. glabripennis* continue to be intercepted along trade pathways (Wu et al. 2017; Krishnankutty et al. 2020). Although high‐risk wood packaging material is targeted for inspection, live insects are still found in ISPM‐15 stamped material (Haack et al. 2014; Greenwood et al. 2023). As we show, knowledge derived from genomic data can elucidate *A. glabripennis* invasion pathways, providing information that can further guide surveillance and management efforts. Our SNP markers can be translated into a target‐enriched screening tool (Altmüller, Budde, and Nürnberg 2014; Diepenbroek et al. 2020) that would be able to rapidly reconstruct the invasion history of new or existing *A. glabripennis* populations or for intercepted individuals. With such a tool, we can also re‐examine global invasion scenarios for other *A. glabripennis* populations (Javal et al. 2019; Lee, Lee, and Lee 2020) to refine our understanding of the invasion history of this important pest. By harnessing the power of genomic data and our refined understanding of *A. glabripennis* invasions, we pave the way toward innovative genomic biosurveillance (Bilodeau et al. 2019) and evidence‐based management strategies that will reduce the risk posed by this invasive pest.

## Conflicts of Interest

The authors declare no conflicts of interest.

## Supporting information


Appendix S1.

Table S1.

## Data Availability

The resources supporting the findings of this study are openly available. The individual genotype data are deposited in the Dryad Digital Repository and can be accessed via the Dryad https://doi.org/10.5061/dryad.280gb5n05. Raw sequence data are available at the Sequence Read Archive (SRA) with the BioProject ID PRJNA824548. The script utilized for analysis is accessible via GitHub at the following URL: https://github.com/mimingcui/invasiveALB.

## References

[eva70036-bib-0001] Abed, A. , G. Legare , S. Pomerleau , J. St‐Cyr , B. Boyle , and F. J. Belzile . 2019. “Genotyping‐By‐Sequencing on the Ion Torrent Platform in Barley.” Methods in Molecular Biology 1900: 233–252. 10.1007/978-1-4939-8944-7_15. 30460569

[eva70036-bib-0002] Akita, A. , T. Katô , T. Yanagi , and K. Kubota . 2021. “Reports of the Alien Species *Anoplophora glabripennis* (Motschulsky, 1853) (Coleoptera, Cerambycidae) Found in Hyogo Pref., Japan.” Gekkan‐Mushi 601: 41–45.

[eva70036-bib-0003] Alexander, D. H. , J. Novembre , and K. Lange . 2009. “Fast Model‐Based Estimation of Ancestry in Unrelated Individuals.” Genome Research 19, no. 9: 1655–1664. 10.1101/gr.094052.109. 19648217 PMC2752134

[eva70036-bib-0004] Altmüller, J. , B. S. Budde , and P. Nürnberg . 2014. “Enrichment of Target Sequences for Next‐Generation Sequencing Applications in Research and Diagnostics.” Biological Chemistry 395, no. 2: 231–237. 10.1515/hsz-2013-0199. 24013102

[eva70036-bib-0005] Aukema, J. E. , B. Leung , K. Kovacs , et al. 2011. “Economic Impacts of Non‐native Forest Insects in the Continental United States.” PLoS One 6, no. 9: e24587. 10.1371/journal.pone.0024587. 21931766 PMC3170362

[eva70036-bib-0006] Aukema, J. E. , D. G. McCullough , B. V. O. N. Holle , and A. M. Liebhold . 2010. “Historical Accumulation of Nonindigenous Forest Pests in the Continental United States.” Bioscience 60, no. 11: 886–897. 10.1525/hio.2010.60.11.5.

[eva70036-bib-0007] Bertelsmeier, C. 2021. “Globalization and the Anthropogenic Spread of Invasive Social Insects.” Current Opinion in Insect Science 46: 16–23. 10.1016/j.cois.2021.01.006. 33545436

[eva70036-bib-0008] Bilodeau, P. , A. D. Roe , G. Bilodeau , et al. 2019. “Biosurveillance of Forest Insects: Part II—Adoption of Genomic Tools by End User Communities and Barriers to Integration.” Journal of Pest Science 92, no. 1: 71–82. 10.1007/s10340-018-1001-1.

[eva70036-bib-0009] Blackburn, G. S. , P. Bilodeau , T. Cooke , et al. 2020. “An Applied Empirical Framework for Invasion Science: Confronting Biological Invasion Through Collaborative Research Aimed at Tool Production.” Annals of the Entomological Society of America 113, no. 4: 230–245. 10.1093/aesa/saz072.

[eva70036-bib-0010] Blackburn, T. M. , P. Pyšek , S. Bacher , et al. 2011. “A Proposed Unified Framework for Biological Invasions.” Trends in Ecology & Evolution 26, no. 7: 333–339. 10.1016/j.tree.2011.03.023. 21601306

[eva70036-bib-0011] Blumenfeld, A. J. , P. A. Eyer , C. Husseneder , et al. 2021. “Bridgehead Effect and Multiple Introductions Shape the Global Invasion History of a Termite.” Communications Biology 4, no. 1: 196. 10.1038/s42003-021-01725-x. 33580197 PMC7881189

[eva70036-bib-0012] Bybee, L. F. , J. G. Millar , T. D. Paine , K. Campbell , and C. C. Hanlon . 2004. “Effects of Temperature on Fecundity and Longevity of *Phoracantha recurva* and *P. semipunctata* (Coleoptera: Cerambycidae).” Environmental Entomology 33: 138–146.

[eva70036-bib-0013] Carter, M. , M. Smith , J. Turgeon , and R. Harrison . 2009. “Analysis of Genetic Diversity in an Invasive Population of Asian Long‐Horned Beetles in Ontario, Canada.” Canadian Entomologist 141, no. 6: 582–594. 10.4039/n09-026.

[eva70036-bib-0014] Carter, M. E. , M. T. Smith , and R. Harrison . 2010. “Genetic Analyses of the Asian Longhorned Beetle (Coleoptera, Cerambycidae, *Anoplophora glabripennis*), in North America, Europe and Asia.” Biological Invasions 12, no. 5: 1165–1182. 10.1007/s10530-009-9538-9.

[eva70036-bib-0015] Carter, M. E. , M. T. Smith , and R. G. Harrison . 2009. “Patterns of Genetic Variation Among Populations of the Asian Longhorned Beetle (Coleoptera: Cerambycidae) in China and Korea.” Annals of the Entomological Society of America 102, no. 5: 895–905. 10.1603/008.102.0516.

[eva70036-bib-0016] Catchen, J. M. , P. A. Hohenlohe , S. Bassham , A. Amores , and W. A. Cresko . 2013. “Stacks: An Analysis Tool Set for Population Genomics.” Molecular Ecology 22, no. 11: 3124–3140. 10.1111/mec.12354. 23701397 PMC3936987

[eva70036-bib-0017] Chang, C. C. , C. C. Chow , L. C. Tellier , S. Vattikuti , S. M. Purcell , and J. J. Lee . 2015. “Second‐Generation PLINK: Rising to the Challenge of Larger and Richer Datasets.” GigaScience 4: 7. 10.1186/s13742-015-0047-8. 25722852 PMC4342193

[eva70036-bib-0018] Ciosi, M. , N. J. Miller , K. S. Kim , R. Giordano , A. Estoup , and T. Guillemaud . 2008. “Invasion of Europe by the Western Corn Rootworm, *Diabrotica virgifera*: Multiple Transatlantic Introductions With Various Reductions of Genetic Diversity.” Molecular Ecology 17, no. 16: 3614–3627. 10.1111/j.1365-294X.2008.03866.x. 18662220

[eva70036-bib-0019] Clavero, M. , and E. Garcia‐Berthou . 2005. “Invasive Species Are a Leading Cause of Animal Extinctions.” Trends in Ecology & Evolution 20, no. 3: 110. 10.1016/j.tree.2005.01.003. 16701353

[eva70036-bib-0020] Collin, F. D. , G. Durif , L. Raynal , et al. 2021. “Extending Approximate Bayesian Computation With Supervised Machine Learning to Infer Demographic History From Genetic Polymorphisms Using DIYABC Random Forest.” Molecular Ecology Resources 21, no. 8: 2598–2613. 10.1111/1755-0998.13413. 33950563 PMC8596733

[eva70036-bib-0021] Cornuet, J. M. , P. Pudlo , J. Veyssier , et al. 2014. “DIYABC v2.0: A Software to Make Approximate Bayesian Computation Inferences About Population History Using Single Nucleotide Polymorphism, DNA Sequence and Microsatellite Data.” Bioinformatics 30, no. 8: 1187–1189. 10.1093/bioinformatics/btt763. 24389659

[eva70036-bib-0022] Coyle, D. R. , R. T. Trotter , M. S. Bean , S. E. Pfister , and N. Walker . 2021. “First Recorded Asian Longhorned Beetle (Coleoptera: Cerambycidae) Infestation in the Southern United States.” Journal of Integrated Pest Management 12, no. 1: 10. 10.1093/jipm/pmab007.

[eva70036-bib-0023] Cui, M. , Y. Wu , M. Javal , et al. 2022. “Genome‐Scale Phylogeography Resolves the Native Population Structure of the Asian Longhorned Beetle, *Anoplophora glabripennis* (Motschulsky).” Evolutionary Applications 15: 934–953. 10.1111/eva.13381. 35782014 PMC9234632

[eva70036-bib-0024] Danecek, P. , A. Auton , G. Abecasis , et al. 2011. “The Variant Call Format and VCFtools.” Bioinformatics 27, no. 15: 2156–2158. 10.1093/bioinformatics/btr330. 21653522 PMC3137218

[eva70036-bib-0025] de Ronne, M. , G. Légaré , F. Belzile , B. Boyle , and D. Torkamaneh . 2023. “3D‐GBS: A Universal Genotyping‐By‐Sequencing Approach for Genomic Selection and Other High‐Throughput Low‐Cost Applications in Species With Small to Medium‐Sized Genomes.” Plant Methods 19: 13. 10.1186/s13007-023-00990-7. 36740716 PMC9899395

[eva70036-bib-0026] Diepenbroek, M. , B. Bayer , K. Schwender , et al. 2020. “Evaluation of the Ion AmpliSeq PhenoTrivium Panel: MPS‐Based Assay for Ancestry and Phenotype Predictions Challenged by Casework Samples.” Genes 11, no. 12: 1398. 10.3390/genes11121398. 33255693 PMC7760956

[eva70036-bib-0027] Dlugosch, K. M. , S. R. Anderson , J. Braasch , F. A. Cang , and H. D. Gillette . 2015. “The Devil Is in the Details: Genetic Variation in Introduced Populations and Its Contributions to Invasion.” Molecular Ecology 24, no. 9: 2095–2111. 10.1111/mec.13183. 25846825

[eva70036-bib-0028] Dlugosch, K. M. , and I. M. Parker . 2008. “Founding Events in Species Invasions: Genetic Variation, Adaptive Evolution, and the Role of Multiple Introductions.” Molecular Ecology 17, no. 1: 431–449. 10.1111/j.1365-294X.2007.03538.x. 17908213

[eva70036-bib-0029] Dodds, K. J. , and D. A. Orwig . 2011. “An Invasive Urban Forest Pest Invades Natural Environments – Asian Longhorned Beetle in Northeastern US Hardwood Forests.” Canadian Journal of Forest Research 41, no. 9: 1729–1742. 10.1139/x11-097.

[eva70036-bib-0030] Dogantzis, K. A. , R. Raffiudin , R. E. Putra , et al. 2024. “Post‐Invasion Selection Acts on Standing Genetic Variation Despite a Severe Founding Bottleneck.” Current Biology 34, no. 6: 1349–1356. 38428415 10.1016/j.cub.2024.02.010

[eva70036-bib-0031] Dupuis, J. R. , S. B. Sim , M. San Jose , et al. 2018. “Population Genomics and Comparisons of Selective Signatures in Two Invasions of Melon Fly, *Bactrocera cucurbitae* (Diptera: Tephritidae).” Biological Invasions 20, no. 5: 1211–1228. 10.1007/s10530-017-1621-z.

[eva70036-bib-0032] Elshire, R. J. , J. C. Glaubitz , Q. Sun , et al. 2011. “A Robust, Simple Genotyping‐By‐Sequencing (GBS) Approach for High Diversity Species.” PLoS One 6, no. 5: e19379. 10.1371/journal.pone.0019379. 21573248 PMC3087801

[eva70036-bib-0033] Epanchin‐Niell, R. S. , and A. M. Liebhold . 2015. “Benefits of Invasion Prevention: Effect of Time Lags, Spread Rates, and Damage Persistence.” Ecological Economics 116: 146–153. 10.1016/j.ecolecon.2015.04.014.

[eva70036-bib-0034] Eschtruth, A. K. , R. A. Evans , and J. J. Battles . 2013. “Patterns and Predictors of Survival in *Tsuga canadensis* Populations Infested by the Exotic Pest *Adelges tsugae*: 20 Years of Monitoring.” Forest Ecology and Management 305: 195–203. 10.1016/j.foreco.2013.05.047.

[eva70036-bib-0035] Estoup, A. , V. Ravigné , R. A. Hufbauer , R. Vitalis , M. Gautier , and B. Facon . 2016. “Is There a Genetic Paradox of Biological Invasion?” Annual Review of Ecology, Evolution, and Systematics 47, no. 1: 51–72. 10.1146/annurev-ecolsys-121415-032116.

[eva70036-bib-0036] Eyer, P. , A. J. Blumenfeld , L. N. L. Johnson , et al. 2021. “Extensive Human‐Mediated Jump Dispersal Within and Across the Native and Introduced Ranges of the Invasive Termite *Reticulitermes flavipes* .” Molecular Ecology 30, no. 16: 3948–3964. 10.1111/mec.16022. 34142394

[eva70036-bib-0037] Eyre, D. , and J. Barbrook . 2021. “The Eradication of Asian Longhorned Beetle at Paddock Wood, UK.” CABI Agriculture and Bioscience 2, no. 1: 12. 10.1186/s43170-021-00034-x.

[eva70036-bib-0038] Fournier, R. E. , and J. J. Turgeon . 2017. “Surveillance During Monitoring Phase of an Eradication Programme Against *Anoplophora glabripennis* (Motschulsky) Guided by a Spatial Decision Support System.” Biological Invasions 19, no. 10: 3013–3035. 10.1007/s10530-017-1505-2.

[eva70036-bib-0039] Garnas, J. R. , M.‐A. Auger‐Rozenberg , A. Roques , et al. 2016. “Complex Patterns of Global Spread in Invasive Insects: Eco‐Evolutionary and Management Consequences.” Biological Invasions 18, no. 4: 935–952. 10.1007/s10530-016-1082-9.

[eva70036-bib-0040] Greenwood, L. F. , D. R. Coyle , M. E. Guerrero , et al. 2023. “Exploring Pest Mitigation Research and Management Associated With the Global Wood Packaging Supply Chain: What and Where Are the Weak Links?” Biological Invasions 25, no. 8: 2395–2421. 10.1007/s10530-023-03058-8.

[eva70036-bib-0041] Haack, R. A. 2006. “Exotic Bark‐ and Wood‐Boring Coleoptera in the United States: Recent Establishments and Interceptions.” Canadian Journal of Forest Research 36, no. 2: 269–288. 10.1139/x05-249.

[eva70036-bib-0042] Haack, R. A. , K. O. Britton , E. G. Brockerhoff , et al. 2014. “Effectiveness of the International Phytosanitary Standard ISPM No. 15 on Reducing Wood Borer Infestation Rates in Wood Packaging Material Entering the United States.” PLoS One 9, no. 5: e96611. 10.1371/journal.pone.0096611. 24827724 PMC4020780

[eva70036-bib-0043] Haack, R. A. , J. F. Cavey , E. R. Hoebeke , and K. Law . 1996. “ *Anoplophora glabripennis*: A New Tree‐Infesting Exotic Cerambycid Invades New York.” Newsletter of the Michigan Entomological Society 41, no. 2–3: 1–3.

[eva70036-bib-0044] Haack, R. A. , F. Hérard , J. Sun , and J. J. Turgeon . 2010. “Managing Invasive Populations of Asian Longhorned Beetle and Citrus Longhorned Beetle: A Worldwide Perspective.” Annual Review of Entomology 55: 521–546. 10.1146/annurev-ento-112408-085427. 19743916

[eva70036-bib-0045] Haack, R. A. , K. R. Law , V. C. Mastro , H. S. Ossenbruggen , and B. J. Raimo . 1997. “New York's Battle With the Asian Long‐Horned Beetle.” Journal of Forestry 95, no. 12: 11–15.

[eva70036-bib-0046] Hamelin, R. C. , and A. D. Roe . 2020. “Genomic Biosurveillance of Forest Invasive Alien Enemies: A Story Written in Code.” Evolutionary Applications 13, no. 1: 95–115. 10.1111/eva.12853. 31892946 PMC6935587

[eva70036-bib-0047] Herms, D. A. , and D. G. McCullough . 2014. “Emerald Ash Borer Invasion of North America: History, Biology, Ecology, Impacts, and Management.” Annual Review of Entomology 59: 13–30. 10.1146/annurev-ento-011613-162051. 24112110

[eva70036-bib-0048] Holmes, T. P. , J. E. Aukema , B. Von Holle , A. M. Liebhold , and E. Sills . 2009. “Economic Impacts of Invasive Species in Forests: Past, Present, and Future.” Annals of the New York Academy of Sciences 1162: 18–38. 10.1111/j.1749-6632.2009.04446.x. 19432643

[eva70036-bib-0049] Hu, J. , S. Angeli , S. Schuetz , Y. Luo , and A. E. Hajek . 2009. “Ecology and Management of Exotic and Endemic Asian Longhorned Beetle *Anoplophora glabripennis* .” Agricultural and Forest Entomology 11, no. 4: 359–375. 10.1111/j.1461-9563.2009.00443.x.

[eva70036-bib-0050] Huang, J. , X. Lu , H. Liu , and S. Zong . 2021. “The Driving Forces of *Anoplophora glabripennis* Have Spatial Spillover Effects.” Forests 12, no. 12: 1678. 10.3390/f12121678.

[eva70036-bib-0051] Hull‐Sanders, H. , E. Pepper , K. Davis , and R. T. Trotter . 2017. “Description of an Establishment Event by the Invasive Asian Longhorned Beetle (*Anoplophora glabripennis*) in a Suburban Landscape in the Northeastern United State.” PLoS One 12: e0181655. 28727772 10.1371/journal.pone.0181655 PMC5519225

[eva70036-bib-0052] Hulme, P. E. 2009. “Trade, Transport and Trouble: Managing Invasive Species Pathways in an Era of Globalization.” Journal of Applied Ecology 46, no. 1: 10–18. 10.1111/j.1365-2664.2008.01600.x.

[eva70036-bib-0053] Javal, M. , E. Lombaert , T. Tsykun , et al. 2019. “Deciphering the Worldwide Invasion of the Asian Longhorned Beetle: A Recurrent Invasion Process From the Native Area Together With a Bridgehead Effect.” Molecular Ecology 28, no. 5: 951–967. 10.1111/mec.15030. 30672635

[eva70036-bib-0054] Javal, M. , A. Roques , J. Haran , F. Hérard , M. Keena , and G. Roux . 2017. “Complex Invasion History of the Asian Long‐Horned Beetle: Fifteen Years After First Detection in Europe.” Journal of Pest Science 92, no. 1: 173–187. 10.1007/s10340-017-0917-1.

[eva70036-bib-0055] Javal, M. , G. Roux , A. Roques , and D. Sauvard . 2018. “Asian Longhorned Beetle Dispersal Potential Assessed in Computer‐Linked Flight‐Mills.” Journal of Applied Entomology 142: 282–286. 10.1111/jen.12408.

[eva70036-bib-0056] Jombart, T. 2008. “Adegenet: A R Package for the Multivariate Analysis of Genetic Markers.” Bioinformatics 24: 1403–1405. 18397895 10.1093/bioinformatics/btn129

[eva70036-bib-0057] Jombart, T. , S. Devillard , and F. Balloux . 2010. “Discriminant Analysis of Principal Components: A New Method for the Analysis of Genetically Structured Populations.” BMC Genetics 11: 94. 10.1186/1471-2156-11-94. 20950446 PMC2973851

[eva70036-bib-0058] Joshi, N. 2011. “Sabre: A Barcode Demultiplexing and Trimming Tool for FastQ Files.” *GitHub*. https://github.com/najoshi/sabre.

[eva70036-bib-0059] Kaňuch, P. , A. Cassel‐Lundhagen , S. Preuss , G. Nordlander , and Å. Berggren . 2022. “Parapatric Genetic Lineages Persist in a Multiply Introduced Non‐native Bush‐Cricket.” Frontiers in Ecology and Evolution 10: 812079. 10.3389/fevo.2022.812079.

[eva70036-bib-0060] Keena, M. A. 2002. “ *Anoplophora glabripennis* (Coleoptera: Cerambycidae) Fecundity and Longevity Under Laboratory Conditions: Comparison of Populations From New York and Illinois on *Acer saccharum* .” Environmental Entomology 31, no. 3: 490–498. 10.1603/0046-225X-31.3.490.

[eva70036-bib-0061] Kim, H. , S. Kim , Y. Lee , H. S. Lee , S. J. Lee , and J. H. Lee . 2021. “Tracing the Origin of Korean Invasive Populations of the Spotted Lanternfly, *Lycorma delicatula* (Hemiptera: Fulgoridae).” Insects 12, no. 6: 539. 10.3390/insects12060539. 34200556 PMC8227202

[eva70036-bib-0062] Kolbe, J. J. , R. E. Glor , L. R. Schettino , A. C. Lara , A. Larson , and J. B. Losos . 2004. “Genetic Variation Increases During Biological Invasion by a Cuban Lizard.” Nature 431, no. 7005: 177–181. 10.1038/nature02807. 15356629

[eva70036-bib-0063] Krehan, H. 2002. “Asian Longhorned Beetle in Austria: Critical Comments on Phytosanitary Measures and Regulations.” In Proceedings of the U.S. Department of Agriculture Interagency Research Forum on Gypsy Moth and Other Invasive Species 2002, edited by S. L. C. Fosbroke and K. W. Gottschalk , 5–6. Newtown Square, PA: U.S. Department of Agriculture, Forest Service, Northeastern Research Station.

[eva70036-bib-0064] Krishnankutty, S. , H. Nadel , A. M. Taylor , et al. 2020. “Identification of Tree Genera Used in the Construction of Solid Wood‐Packaging Materials That Arrived at U.S. Ports Infested With Live Wood‐Boring Insects.” Journal of Economic Entomology 113, no. 3: 1183–1194. 10.1093/jee/toaa060. 32304325

[eva70036-bib-0065] Ladin, Z. S. , D. A. Eggen , T. L. E. Trammell , and V. D'Amico . 2023. “Human‐Mediated Dispersal Drives the Spread of the Spotted Lanternfly (*Lycorma delicatula*).” Scientific Reports 13, no. 1: 1098. 10.1038/s41598-022-25989-3. 36658159 PMC9852583

[eva70036-bib-0066] Lee, S. , Y. Lee , and S. Lee . 2020. “Population Genetic Structure of *Anoplophora glabripennis* in South Korea: Invasive Populations in the Native Range?” Journal of Pest Science 93: 1181–1196. 10.1007/s10340-020-01245-3.

[eva70036-bib-0067] Li, H. 2013. “Aligning Sequence Reads, Clone Sequences and Assembly Contigs With BWA‐MEM.” arXiv Preprint, arXiv: 1303.3997. 10.48550/arXiv.1303.3997.

[eva70036-bib-0068] Li, H. , B. Handsaker , A. Wysoker , et al. 2009. “The Sequence Alignment/Map Format and SAMtools.” Bioinformatics 25, no. 16: 2078–2079. 10.1093/bioinformatics/btp352. 19505943 PMC2723002

[eva70036-bib-0069] Liebhold, A. M. , L. Berec , E. G. Brockerhoff , et al. 2016. “Eradication of Invading Insect Populations: From Concepts to Applications.” Annual Review of Entomology 61: 335–352. 10.1146/annurev-ento-010715-023809. 26667377

[eva70036-bib-0070] Lingafelter, S. W. , and E. R. Hoebeke . 2002. Revision of the Genus Anoplophora (Coleoptera: Cerambycidae), edited by D. R. Smith . Washington, DC: Entomological Society of Washington.

[eva70036-bib-0071] Liu, Y. , J. Henkel , A. Beaurepaire , J. D. Evans , P. Neumann , and Q. Huang . 2021. “Comparative Genomics Suggests Local Adaptations in the Invasive Small Hive Beetle.” Ecology and Evolution 11, no. 22: 15780–15791. 10.1002/ece3.8242. 34824789 PMC8601931

[eva70036-bib-0072] Lockwood, J. L. , P. Cassey , and T. M. Blackburn . 2009. “The More You Introduce the More You Get: The Role of Colonization Pressure and Propagule Pressure in Invasion Ecology.” Diversity and Distributions 15, no. 5: 904–910. 10.1111/j.1472-4642.2009.00594.x.

[eva70036-bib-0073] Lombaert, E. , T. Guillemaud , J. M. Cornuet , T. Malausa , B. Facon , and A. Estoup . 2010. “Bridgehead Effect in the Worldwide Invasion of the Biocontrol Harlequin Ladybird.” PLoS One 5, no. 3: e9743. 10.1371/journal.pone.0009743. 20305822 PMC2840033

[eva70036-bib-0074] Lopez, V. M. , M. S. Hoddle , J. A. Francese , D. R. Lance , and A. M. Ray . 2017. “Assessing Flight Potential of the Invasive Asian Longhorned Beetle (Coleoptera: Cerambycidae) With Computerized Flight Mills.” Journal of Economic Entomology 110: 1070–1077. 10.1093/jee/tox0. 28419382

[eva70036-bib-0075] Loxdale, H. D. 2010. “Rapid Genetic Changes in Natural Insect Populations.” Ecological Entomology 35: 155–164. 10.1111/j.1365-2311.2009.01141.x.

[eva70036-bib-0076] Lu, W. , Q. Wang , M. Y. Tian , et al. 2013. “Reproductive Traits of *Glenea cantor* (Coleoptera: Cerambycidae: Lamiinae).” Journal of Economic Entomology 106, no. 1: 215–220. 23448034 10.1603/ec12251

[eva70036-bib-0077] Luo, Y. , J. Wen , and Z. Xu . 2003. “Current Situation of Research and Control on Poplar Longhorned Beetle, Especially for *Anoplophora glabripennis* in China.” Nachrichtenblatt des Deutschen Pflanzenschutzdienstes 55: 66–67.

[eva70036-bib-0078] Mainka, S. A. , and G. W. Howard . 2010. “Climate Change and Invasive Species: Double Jeopardy.” Integrative Zoology 5, no. 2: 102–111. 10.1111/j.1749-4877.2010.00193.x. 21392328

[eva70036-bib-0079] Manichaikul, A. , J. C. Mychaleckyj , S. S. Rich , K. Daly , M. Sale , and W. M. Chen . 2010. “Robust Relationship Inference in Genome‐Wide Association Studies.” Bioinformatics 26, no. 22: 2867–2873. 10.1093/bioinformatics/btq559. 20926424 PMC3025716

[eva70036-bib-0080] Martin, M. 2011. “Cutadapt Removes Adapter Sequences From High‐Throughput Sequencing Reads.” EMBnet Journal 17: 10.

[eva70036-bib-0081] McCulloch, G. A. , and J. M. Waters . 2023. “Rapid Adaptation in a Fast‐Changing World: Emerging Insights From Insect Genomics.” Global Change Biology 29, no. 4: 943–954. 10.1111/gcb.16512. 36333958 PMC10100130

[eva70036-bib-0082] McKenna, D. D. , E. D. Scully , Y. Pauchet , et al. 2016. “Genome of the Asian Longhorned Beetle (*Anoplophora glabripennis*), a Globally Significant Invasive Species, Reveals Key Functional and Evolutionary Innovations at the Beetle‐Plant Interface.” Genome Biology 17, no. 1: 227. 10.1186/s13059-016-1088-8. 27832824 PMC5105290

[eva70036-bib-0083] Meirmans, P. G. 2014. “Nonconvergence in Bayesian Estimation of Migration Rates.” Molecular Ecology Resources 14, no. 4: 726–733. 10.1111/1755-0998.12216. 24373147

[eva70036-bib-0084] Melbourne, B. A. , and A. Hastings . 2009. “Highly Variable Spread Rates in Replicated Biological Invasions: Fundamental Limits to Predictability.” Science 325, no. 5947: 1536–1539. 10.1126/science.1176138. 19762641

[eva70036-bib-0085] Meng, P. S. , K. Hoover , and M. A. Keena . 2015. “Asian Longhorned Beetle (Coleoptera: Cerambycidae), an Introduced Pest of Maple and Other Hardwood Trees in North America and Europe.” Journal of Integrated Pest Management 6, no. 1: 1–13. 10.1093/jipm/pmv003.

[eva70036-bib-0086] Mussmann, S. M. , M. R. Douglas , T. K. Chafin , M. E. Douglas , and S. Jarman . 2019. “BA3‐SNPs: Contemporary Migration Reconfigured in BayesAss for Next‐Generation Sequence Data.” Methods in Ecology and Evolution 10, no. 10: 1808–1813. 10.1111/2041-210x.13252.

[eva70036-bib-0087] Ortego, J. , V. Cespedes , A. Millan , and A. J. Green . 2021. “Genomic Data Support Multiple Introductions and Explosive Demographic Expansions in a Highly Invasive Aquatic Insect.” Molecular Ecology 30, no. 17: 4189–4203. 10.1111/mec.16050. 34192379

[eva70036-bib-0088] Pejchar, L. , and H. A. Mooney . 2009. “Invasive Species, Ecosystem Services and Human Well‐Being.” Trends in Ecology & Evolution 24, no. 9: 497–504. 10.1016/j.tree.2009.03.016. 19577817

[eva70036-bib-0089] Picq, S. , M. A. Keena , N. P. Havill , et al. 2018. “Assessing the Potential of Genotyping‐By‐Sequencing‐Derived Single Nucleotide Polymorphisms to Identify the Geographic Origins of Intercepted Gypsy Moth (*Lymantria dispar*) Specimens: A Proof‐Of‐Concept Study.” Evolutionary Applications 11, no. 3: 325–339. 10.1111/eva.12559.

[eva70036-bib-0090] Picq, S. , Y. Wu , V. V. Martemyanov , et al. 2023. “Range‐Wide Population Genomics of the Spongy Moth, *Lymantria dispar* (Erebidae): Implications for Biosurveillance, Subspecies Classification and Phylogeography of a Destructive Moth.” Evolutionary Applications 16, no. 3: 638–656. 10.1111/eva.13522. 36969137 PMC10033852

[eva70036-bib-0091] Poland, T. 1998. “Chicago Joins New York in Battle With the Asian Longhorned Beetle.” Newsletter of the Michigan Entomological Society 43: 15–17.

[eva70036-bib-0092] Pudlo, P. , J. M. Marin , A. Estoup , J. M. Cornuet , M. Gautier , and C. P. Robert . 2016. “Reliable ABC Model Choice via Random Forests.” Bioinformatics 32, no. 6: 859–866. 10.1093/bioinformatics/btv684. 26589278

[eva70036-bib-0093] Pyšek, P. , P. E. Hulme , D. Simberloff , et al. 2020. “Scientists' Warning on Invasive Alien Species.” Biological Reviews 95, no. 6: 1511–1534. 10.1111/brv.12627. 32588508 PMC7687187

[eva70036-bib-0094] Pyšek, P. , and D. M. Richardson . 2010. “Invasive Species, Environmental Change and Management, and Health.” Annual Review of Environment and Resources 35, no. 1: 25–55. 10.1146/annurev-environ-033009-095548.

[eva70036-bib-0095] Rassati, D. , R. A. Haack , M. Knizek , and M. Faccoli . 2018. “National Trade Can Drive Range Expansion of Bark‐ and Wood‐Boring Beetles.” Journal of Economic Entomology 111, no. 1: 260–268. 10.1093/jee/tox308. 29272425

[eva70036-bib-0096] Rimmer, A. , H. Phan , I. Mathieson , et al. 2014. “Integrating Mapping‐, Assembly‐ and Haplotype‐Based Approaches for Calling Variants in Clinical Sequencing Applications.” Nature Genetics 46, no. 8: 912–918. 10.1038/ng.3036. 25017105 PMC4753679

[eva70036-bib-0097] Rius, M. , and J. A. Darling . 2014. “How Important Is Intraspecific Genetic Admixture to the Success of Colonising Populations?” Trends in Ecology & Evolution 29, no. 4: 233–242. 10.1016/j.tree.2014.02.003. 24636862

[eva70036-bib-0098] Roe, A. D. , A. S. Torson , G. Bilodeau , et al. 2019. “Biosurveillance of Forest Insects: Part I—Integration and Application of Genomic Tools to the Surveillance of Non‐native Forest Insects.” Journal of Pest Science 92, no. 1: 51–70. 10.1007/s10340-018-1027-4.

[eva70036-bib-0099] Roques, A. , M.‐A. Auger‐Rozenberg , T. M. Blackburn , et al. 2016. “Temporal and Interspecific Variation in Rates of Spread for Insect Species Invading Europe During the Last 200 Years.” Biological Invasions 18, no. 4: 907–920. 10.1007/s10530-016-1080-y.

[eva70036-bib-0100] Sawyer, A. 2007. “Infestation Dynamics of the Asian Longhorned Beetle in the United States.” Paper presented at the Interagency Research Forum on Gypsy Moth and Other Invasive Species, Annapolis, MD.

[eva70036-bib-0101] Schmidt, T. L. , A. R. van Rooyen , J. Chung , et al. 2019. “Tracking Genetic Invasions: Genome‐Wide Single Nucleotide Polymorphisms Reveal the Source of Pyrethroid‐Resistant *Aedes aegypti* (Yellow Fever Mosquito) Incursions at International Ports.” Evolutionary Applications 12, no. 6: 1136–1146. 10.1111/eva.12787. 31297145 PMC6597869

[eva70036-bib-0102] Schmitt, L. 2023. “Phenology of Asian Longhorned Beetle (*Anoplophora Glabripennis*, Coleoptera: Cerambycidae) in South Carolina, USA.” Master's thesis, Clemson University.

[eva70036-bib-0103] Schrieber, K. , and S. Lachmuth . 2017. “The Genetic Paradox of Invasions Revisited: The Potential Role of Inbreeding × Environment Interactions in Invasion Success.” Biological Reviews 92, no. 2: 939–952. 10.1111/brv.12263. 27009691

[eva70036-bib-0104] Sherpa, S. , M. G. B. Blum , T. Capblancq , T. Cumer , D. Rioux , and L. Despres . 2019. “Unravelling the Invasion History of the Asian Tiger Mosquito in Europe.” Molecular Ecology 28, no. 9: 2360–2377. 10.1111/mec.15071. 30849200

[eva70036-bib-0105] Sherpa, S. , J. Renaud , M. Gueguen , et al. 2020. “Landscape Does Matter: Disentangling Founder Effects From Natural and Human‐Aided Post‐Introduction Dispersal During an Ongoing Biological Invasion.” Journal of Animal Ecology 89, no. 9: 2027–2042. 10.1111/1365-2656.13284. 32597498

[eva70036-bib-0106] Short, M. T. , K. D. Chase , T. E. Feeley , A. M. Kees , J. T. Wittman , and B. H. Aukema . 2019. “Rail Transport as a Vector of Emerald Ash Borer.” Agricultural and Forest Entomology 22, no. 1: 92–97. 10.1111/afe.12360.

[eva70036-bib-0107] Simberloff, D. 2009. “The Role of Propagule Pressure in Biological Invasions.” Annual Review of Ecology, Evolution, and Systematics 40, no. 1: 81–102. 10.1146/annurev.ecolsys.110308.120304.

[eva70036-bib-0108] Sjöman, H. , J. Östberg , and J. Nilsson . 2014. “Review of Host Trees for the Wood‐Boring Pests *Anoplophora glabripennis* and *Anoplophora chinensis*: An Urban Forest Perspective.” Arboriculture & Urban Forestry 40: 143–164.

[eva70036-bib-0109] Smith, M. T. , J. Bancroft , G. Li , R. Gao , and S. A. Teale . 2001. “Dispersal of *Anoplophora glabripennis* (Cerambycidae).” Environmental Entomology 30, no. 6: 1036–1040. 10.1603/0046-225X-30.6.1036.

[eva70036-bib-0110] Smith, M. T. , P. C. Tobin , J. Bancroft , G. Li , and R. Gao . 2004. “Dispersal and Spatiotemporal Dynamics of Asian Longhorned Beetle (Coleoptera: Cerambycidae) in China.” Environmental Entomology 33, no. 2: 435–442.

[eva70036-bib-0111] Smith, M. T. , J. J. Turgeon , P. De Groot , and B. Gasman . 2009. “Asian Longhorned Beetle *Anoplophora glabripennis* (Motschulsky): Lessons Learned and Opportunities to Improve the Process of Eradiation and Management.” American Entomologist 55: 21–25.

[eva70036-bib-0112] Stamatakis, A. 2014. “RAxML Version 8: A Tool for Phylogenetic Analysis and Post‐Analysis of Large Phylogenies.” Bioinformatics 30, no. 9: 1312–1313. 10.1093/bioinformatics/btu033. 24451623 PMC3998144

[eva70036-bib-0113] Straw, N. A. , C. Tilbury , N. J. Fielding , D. T. Williams , and T. Cull . 2015. “Timing and Duration of the Life Cycle of Asian Longhorn Beetle *Anoplophora glabripennis* (Coleoptera: Cerambycidae) in Southern England.” Agricultural and Forest Entomology 17, no. 4: 400–411.

[eva70036-bib-0114] Tay, W. T. , R. L. Meagher Jr. , C. Czepak , and A. T. Groot . 2022. “ *Spodoptera frugiperda:* Ecology, Evolution, and Management Options of an Invasive Species.” Annual Review of Entomology 68, no. 1: 299–317. 10.1146/annurev-ento-120220-102548. 36198399

[eva70036-bib-0115] Torkamaneh, D. , J. Laroche , M. Bastien , A. Abed , and F. Belzile . 2017. “Fast‐GBS: A New Pipeline for the Efficient and Highly Accurate Calling of SNPs From Genotyping‐By‐Sequencing Data.” BMC Bioinformatics 18, no. 1: 5. 10.1186/s12859-016-1431-9. 28049422 PMC5210301

[eva70036-bib-0116] Torkamaneh, D. , J. Laroche , and F. Belzile . 2016. “Genome‐Wide SNP Calling From Genotyping by Sequencing (GBS) Data: A Comparison of Seven Pipelines and Two Sequencing Technologies.” PLoS One 11, no. 8: e0161333. 10.1371/journal.pone.0161333. 27547936 PMC4993469

[eva70036-bib-0117] Trotter, R. T., III , and H. M. Hull‐Sanders . 2015. “Quantifying Dispersal of the Asian Longhorned Beetle (*Anoplophora glabripennis*, Coleoptera) With Incomplete Data and Behavioral Knowledge.” Biological Invasions 17, no. 12: 3359–3369. 10.1007/s10530-015-0961-9.

[eva70036-bib-0118] Turbelin, A. J. , R. N. Cuthbert , F. Essl , P. J. Haubrock , A. Ricciardi , and F. Courchamp . 2023. “Biological Invasions Are as Costly as Natural Hazards.” Perspectives in Ecology and Conservation 21, no. 2: 143–150. 10.1016/j.pecon.2023.03.002.

[eva70036-bib-0119] Turgeon, J. J. , B. Gasman , M. T. Smith , et al. 2022. “Canada's Response to Invasion by Asian Longhorned Beetle (Coleoptera: Cerambycidae) in Ontario.” Canadian Entomologist 154, no. s1: e1. 10.4039/tce.2021.60.

[eva70036-bib-0120] Turgeon, J. J. , M. Orr , C. Grant , Y. Wu , and B. Gasman . 2015. “Decade‐Old Satellite Infestation of *Anoplophora glabripennis* Motschulsky (Coleoptera: Cerambycidae) Found in Ontario, Canada Outside Regulated Area of Founder Population.” Oleopterists Bulletin 69, no. 4: 674–678. 10.1649/0010-065x-69.4.674.

[eva70036-bib-0121] Turgeon, J. J. , M. T. Smith , J. H. Pedlar , R. E. Fournier , M. Orr , and B. Gasman . 2021. “Tree Selection and Use by the Polyphagous Xylophage *Anoplophora glabripennis* (Coleoptera: Cerambycidae) in Canada.” Canadian Journal of Forest Research 52, no. 4: 622–643. 10.1139/cjfr-2021-0244.

[eva70036-bib-0122] van Rees, C. B. , B. K. Hand , S. C. Carter , et al. 2022. “A Framework to Integrate Innovations in Invasion Science for Proactive Management.” Biological Reviews 97: 1712–1735. 10.1111/brv.12859. 35451197

[eva70036-bib-0123] Wang, L. , C. Li , Y. Luo , et al. 2023. “Current and Future Control of the Wood‐Boring Pest *Anoplophora glabripennis* .” Insect Science 30, no. 6: 1534–1551. 36944595 10.1111/1744-7917.13187

[eva70036-bib-0124] Whitney, K. D. , and C. A. Gabler . 2008. “Rapid Evolution in Introduced Species, “Invasive Traits” and Recipient Communities: Challenges for Predicting Invasive Potential.” Diversity and Distributions 14: 569–580.

[eva70036-bib-0125] Wilson, G. , and B. Rannala . 2003. “Bayesian Inference of Recent Migration Rates Using Multilocus Genotypes.” Genetics 163: 1177–1191. 12663554 10.1093/genetics/163.3.1177 PMC1462502

[eva70036-bib-0126] Wongnikong, W. , J. P. Hereward , S. L. van Brunschot , and G. H. Walter . 2021. “Multiple Invasions of *Bemisia argentifolii* Into Australia and Its Current Genetic Connectivity Across Space.” Journal of Pest Science 94, no. 4: 1331–1343.

[eva70036-bib-0127] Wu, Y. , N. F. Trepanowski , J. J. Molongoski , et al. 2017. “Identification of Wood‐Boring Beetles (Cerambycidae and Buprestidae) Intercepted in Trade‐Associated Solid Wood Packaging Material Using DNA Barcoding and Morphology.” Scientific Reports 7: 40316. 10.1038/srep40316. 28091577 PMC5238391

[eva70036-bib-0128] Yan, J. 1985. “Study on the Distribution Area of *Anoplophora glabripennis* in East China.” Journal of North‐Eastern Forestry College 13: 63–69.

[eva70036-bib-0129] Yemshanov, D. , R. G. Haight , F. H. Koch , et al. 2017. “Robust Surveillance and Control of Invasive Species Using a Scenario Optimization Approach.” Ecological Economics 133: 86–98. 10.1016/j.ecolecon.2016.11.018.

[eva70036-bib-0130] Zhang, X. , T. Huang , L. Zhang , et al. 2016. “Three‐North Shelter Forest Program Contribution to Long‐Term Increasing Trends of Biogenic Isoprene Emissions in Northern China.” Atmospheric Chemistry and Physics 16, no. 11: 6949–6960. 10.5194/acp-16-6949-2016.

[eva70036-bib-0131] Zhang, X. , J. G. Rayner , M. Blaxter , and N. W. Bailey . 2021. “Rapid Parallel Adaptation Despite Gene Flow in Silent Crickets.” Nature Communications 12, no. 1: 50. 10.1038/s41467-020-20263-4. PMC7782688 33397914

[eva70036-bib-0132] Zhou, J. , K. Zhang , and Y. Lu . 1984. “Study on Adult Activity and Behavioral Mechanism of *Anoplophora nobilis* Ganglbauer.” Scientia Silvae Sinica 20: 372–379.

