# Supplementary material for: Tracking the North American Asian Longhorned Beetle Invasion With Genomics
Source: Evol Appl. 2024 Nov 19;17(11):e70036. doi: 10.1111/eva.70036 (PMC11576519; doi:10.1111/eva.70036)
Supplement: Supplementary file 1 — Appendix S1. [file EVA-17-e70036-s001.docx]

# **Supplementary Methods**

## ***Scenario design for DIYABC***

To investigate the invasion history of North American ALB populations, we followed an 8-step analysis (Figure S8). The optimal scenario for each step is selected based on classification votes for each scenario, which represents the number of times a scenario is selected in a forest of 1000 simulated trees. The scenario with the most classification votes predicted was selected. We limited our analyses to five invasive populations (MA, OH, IL, TOR and Far) due to the limited sample size in NJ and evidence of admixture in NY.

Here, we first tested the relationships among three native populations N1, N2 and NE (Step one). In Step one, we considered three populations with various topologies. The earliest ancestor population (Nanc) is always considered unsampled. When a split occurs, a scenario with an unsampled population is also considered. Scenario nine depicts N1 and NE originating from an unsampled population, which diverged from Nanc, as did N2.

In Step two, we designed the scenarios based on the selected scenario from the previous step and add population NW to the analysis. Scenario four emerged as the optimal one, in which N1 and NW diverged from an unsampled population, which, along with NE, originated from another unsampled population that, with N2, originated from Nanc.

Following the same strategy, we tested Step three by adding population S. In this step, scenario two was selected, where population S originated from an unsampled population along with N2.

From Step four to eight, we tested five (or six for MA) invasion scenarios for invasive populations of IL, TOR, OH, MA, and Far, respectively. We considered that the invasive population originated from an unsampled native population derived from each sampled native population, respectively. Following the introduction, a bottleneck event was considered since it is a common process during invasion. For population MA, we included one mixed ancestor scenario, which assumes MA originated from the admixture of N2 and S, given that our results show MA is genetically close to both native populations (Figure 3).

## ***Prior parameters***

We considered two types of prior parameters, population size (N) and time (T). N includes effective population size for populations at the time of sampling and the population size of the invasive founder population, while T includes divergence time point and bottleneck duration. We kept the prior parameters of the effective population size of the native populations (including unsampled ones) with a broad range of 10-10,000 set as default (Table S6). The divergence times for populations were found to range from thousands of years such as in the dung beetle *Phelotrupes auratus* populations (Araki & Sota. (2022), to more ancient times. For example, three major lineages of *Ducetia japonica* were found formed before the Last Glacial Maximum (Zhou et al., 2021). Here, we assumed the divergence time of the native populations to range from 10 to 3,000, following the DIYABC analyses conducted by Javal *et al.* (2019), where the prior for the native populations was set to 200-2000. For invasive populations, we chose a smaller range for the effective population size of the founder populations as 10-100. After the bottleneck, we do not expect a significant increase in effective population size; however, during the invasion process, there may be factors such as mutations that could increase population diversity, thus increase the effective population size. Therefore, we set a size of 10-1,000 for the post-bottleneck population expansion. For the prior intervals of the introduction date and bottleneck duration, we assumed a single generation per year since this has been observed in many region (Haack et al., 2010; Hu et al., 2009), although this may vary in parts of the native and invasive range (Coyle et al., 2021; Hu et al., 2009; Straw et al., 2015). We assumed a single introduction in each invasive location in our tested scenarios based on our STRUCTURE and DAPC results (Figures 1-3). Therefore, the priors for invasion events were defined based on the first sampling year. We adjusted the prior interval accordingly based on their first official record and our first sampling date, with taking account of a lag phase of 20 years based on Morimoto’s finding that lag time takes 4.4-23.2 years for the invasive insects they studied (Morimoto et al., 2019) (See Table S6 for details).

**References**

Araki, Y., & Sota, T. (2023). Whole‐genome resequencing reveals recent divergence of geographic populations of the dung beetle *Phelotrupes auratus* with color variation. *Ecology and Evolution*, *13*(1), e9765.

Coyle, D. R., Trotter, R. T., Bean, M. S., & Pfister, S. E. (2021). First Recorded Asian Longhorned Beetle (Coleoptera: Cerambycidae) Infestation in the Southern United States. *Journal of Integrated Pest Management*, *12*(1). <https://doi.org/10.1093/jipm/pmab007>

Haack, R. A., Hérard, F., Sun, J., & Turgeon, J. J. (2010). Managing invasive populations of Asian longhorned beetle and citrus longhorned beetle: A worldwide perspective. *Annual Review of Entomology*, *55*, 521–546. <https://doi.org/10.1146/annurev-ento-112408-085427>

Javal, M., Lombaert, E., Tsykun, T., Courtin, C., Kerdelhue, C., Prospero, S., . . . Roux, G. (2019). Deciphering the worldwide invasion of the Asian longhorned beetle: a recurrent invasion process from the native area together with a bridgehead effect. *Molecular Ecology, 28*(5), 951-967. doi:10.1111/mec.15030

Hu, J., Angeli, S., Schuetz, S., Luo, Y., & Hajek, A. E. (2009). Ecology and management of exotic and endemic Asian longhorned beetle *Anoplophora glabripennis*. *Agricultural and Forest Entomology*, *11*, 359–375. <https://doi.org/10.1111/j.1461-9563.2009.00443.x>

Morimoto, N., Kiritani, K., Yamamura, K., & Yamanaka, T. (2019). Finding indications of lag time, saturation and trading inflow in the emergence record of exotic agricultural insect pests in Japan. *Applied Entomology and Zoology*, *54*(4), 437–450. <https://doi.org/10.1007/s13355-019-00640-2>

Straw, N. A., Tilbury, C., Fielding, N. J., Williams, D. T., & Cull, T. (2015). Timing and duration of the life cycle of Asian longhorn beetle *Anoplophora glabripennis* (Coleoptera: Cerambycidae) in southern England. *Agricultural and Forest Entomology*, *17*(4), 400–411. <https://doi.org/10.1111/afe.12120>

Zhou, Z. J., Zhen, Y. X., Guan, B., Ma, L., & Wang, W. J. (2021). Phylogeography and genetic diversity of the widespread katydid *Ducetia japonica* (Thunberg, 1815) across China. *Ecology and Evolution*, 11(9), 4276-4294.

#
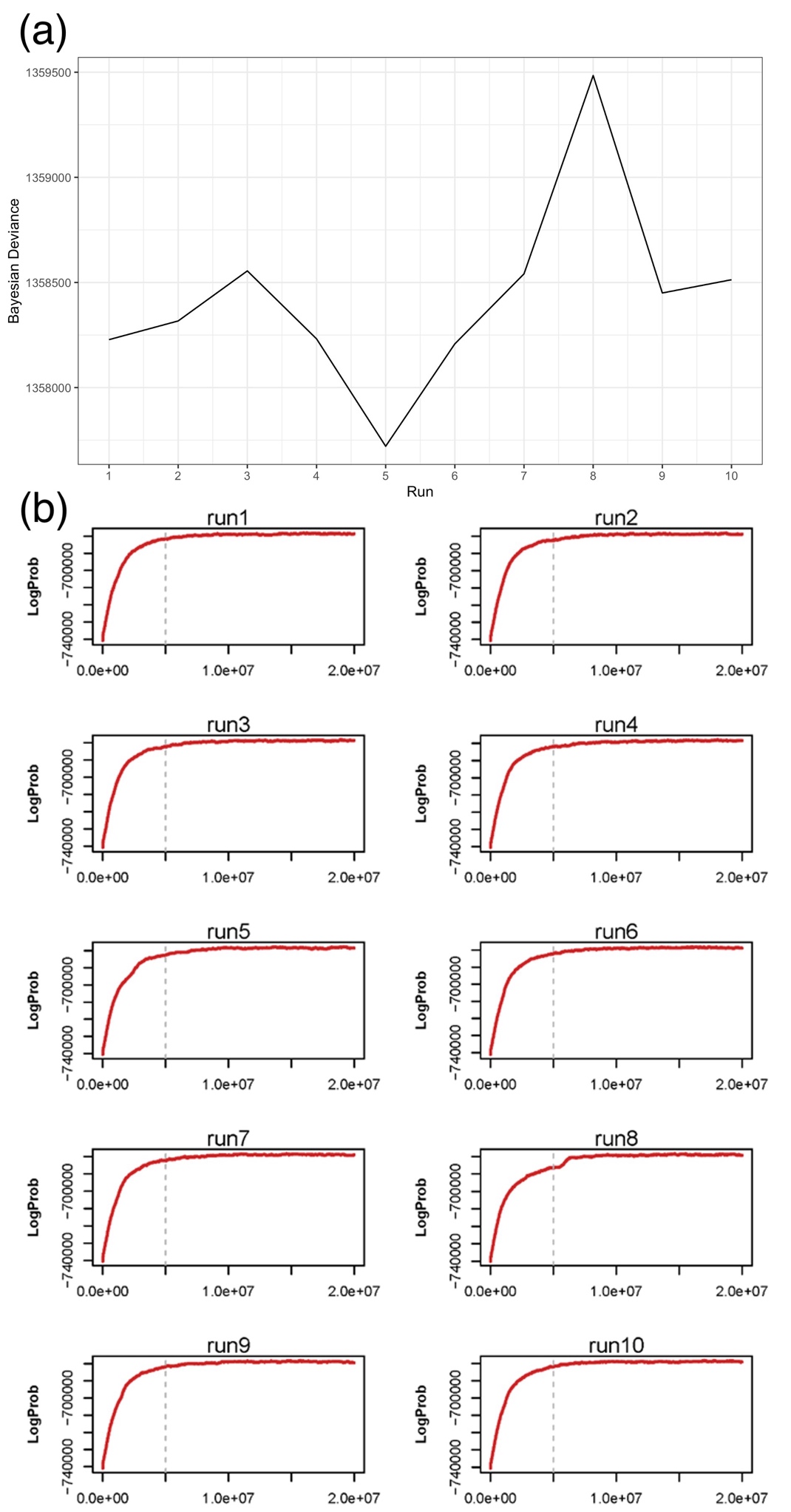
Supplementary Figures

**Figure S1:** Trace plots of ten replicate runs in BayesAss. (a) Bayesian deviance of the ten runs. (b) Horizontal axis is the state of the iterations, with the vertical dashed line indicating the end of the burn-in period (i.e., 5,000,000 iterations). Run five showed the lowest Bayesian deviance and was selected for downstream parameter estimation.


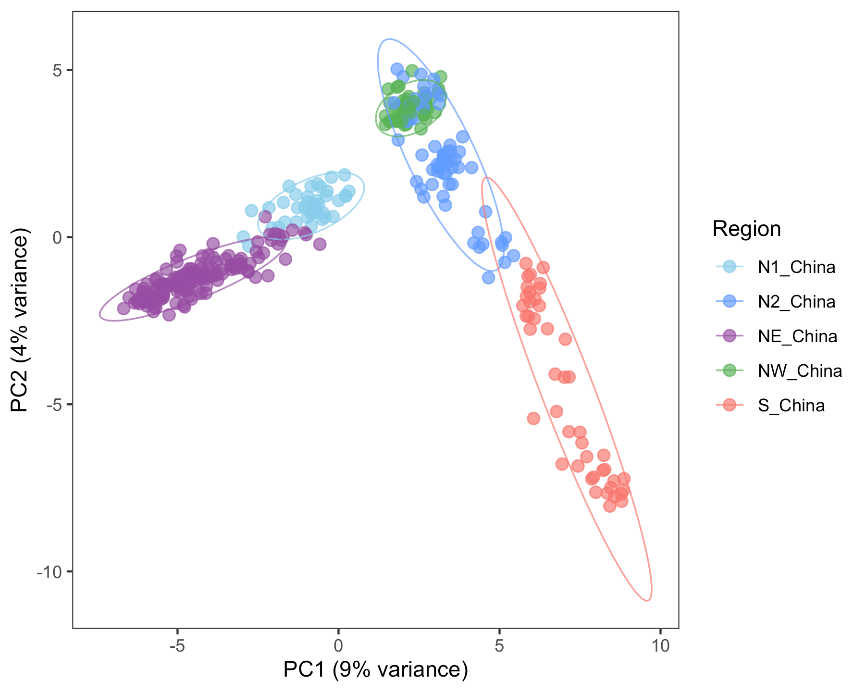


**Figure S2:** Principal component analysis for the native *A. glabripennis* samples. Colored groups represent different regions in China, i.e., N1 (North 1), N2 (North 2), NE (Northeast), NW (Northwest) and S (South) as in Cui et al. (2022).

**
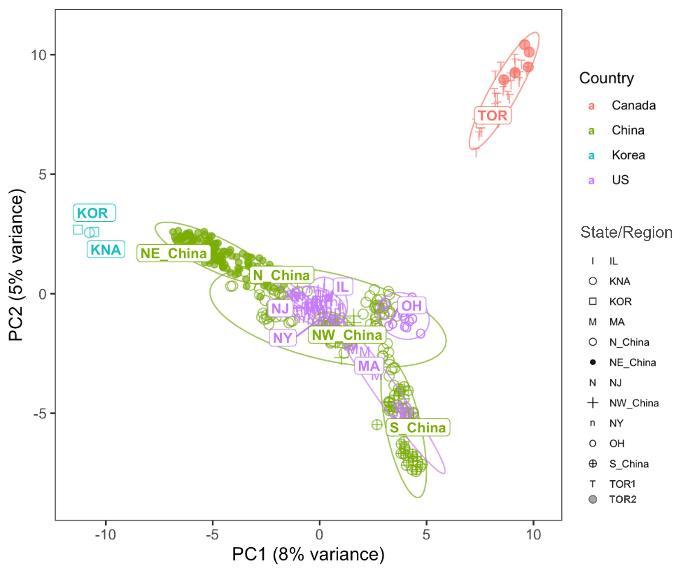
**

**Figure S3** Principal component analysis for global *A. glabripennis* with each country represented by different colors and each population/region represented by separate symbols. Native range: KNA - Pocheon; KOR - Gangwon; NE - Northeast China; NW - Northwest China; N - North China; S - South China. Invasive range: MA - Massachusetts, US; OH - Ohio, US; IL - Illinois, US; NJ - New Jersey, US; NY - New York, US; TOR - Toronto, Canada. TOR1 represented the initial *A. glabripennis* infestation (2003) and TOR2 are specimens from the second find in 2013.


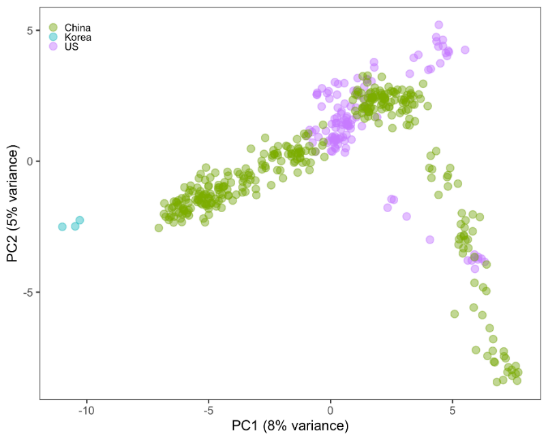


**Figure S4:** Principal component analysis of *A. glabripennis* data set excluding the Toronto samples.


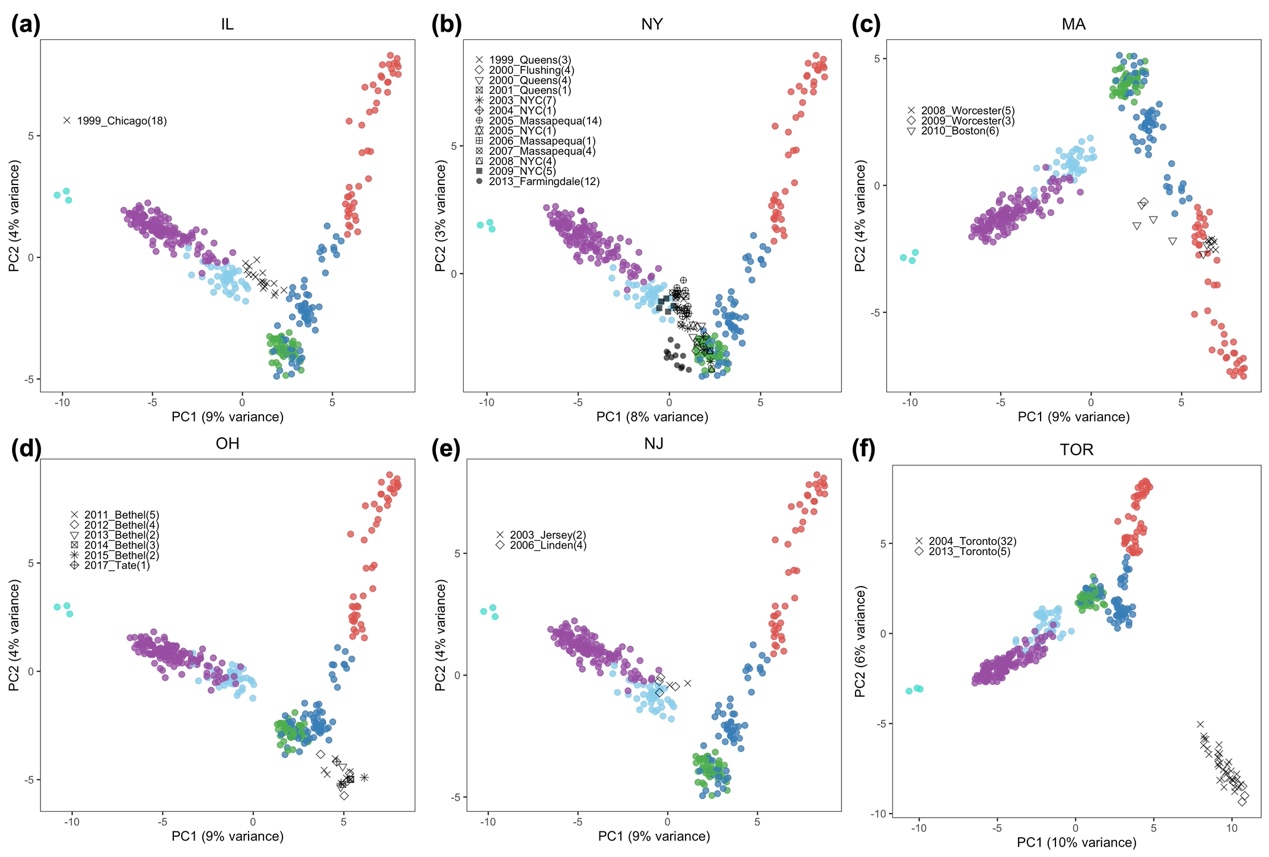


**Figure S5** Principal component analysis for each invasive population (black symbols) with the native *A. glabripennis* reference collection (colored circles). Collection year for each sample is indicated by different symbols. (a) Illinois (1999); (b) New York (1999-2013); (c) Massachusetts (2008-2010); (d) Ohio (2011-2017); (e) New Jersey (2003, 2006); (f) Toronto (2004, 2013). Regions colored as in Figure S2, Korea in turquoise.


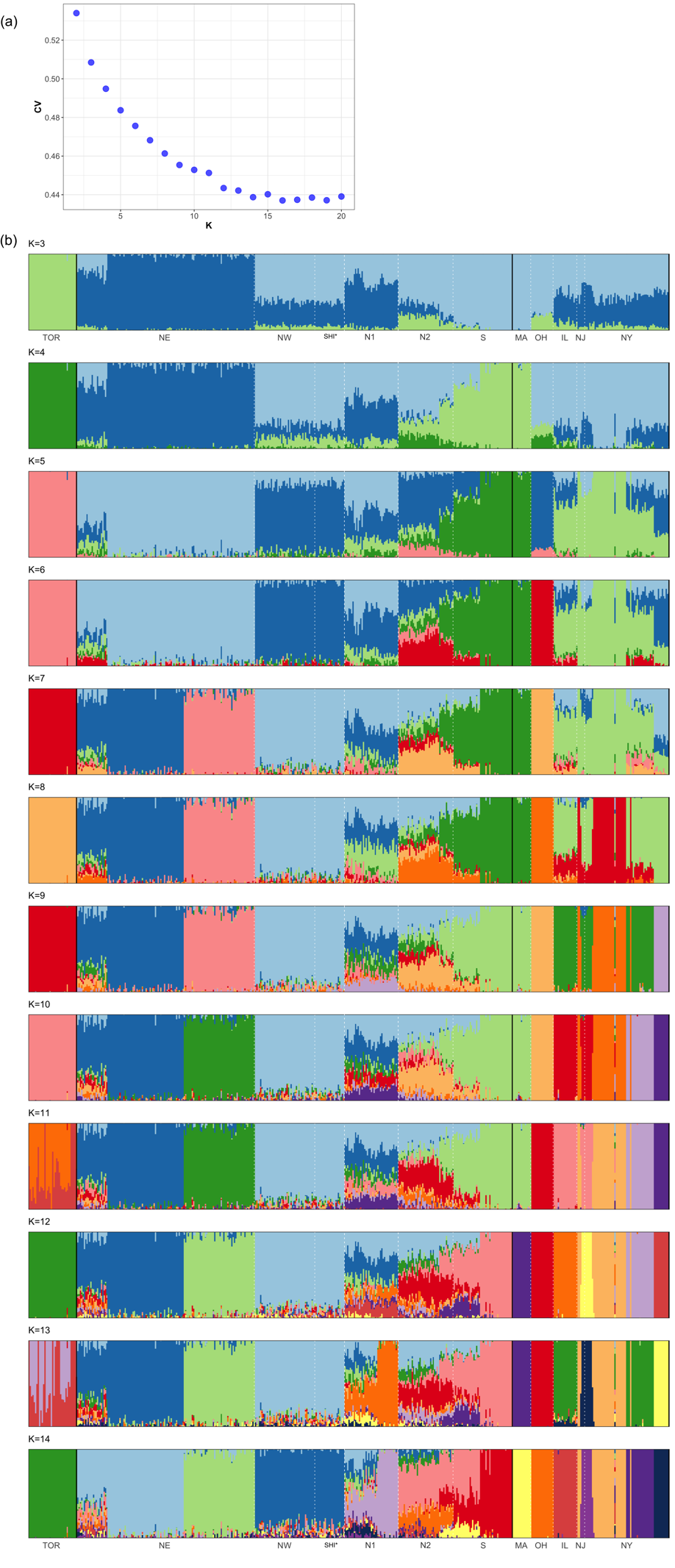


**Figure S6:** Admixture analysis. (a) Cross validation (CV) error plot. X-axis shows numbers of K and y-axis shows CV error. (b) Bar plot when K = 3 to K = 14. Native range areas are as follows: NE - Northeast China; NW - Northwest China; N1 - North China one; N2 – North China two; SHI - Shijiazhuang. S - South China. Invasive range includes TOR - Toronto, Canada; MA - Massachusetts, US; OH - Ohio, US; IL - Illinois, US; NJ - New Jersey, US; NY - New York, US.

*
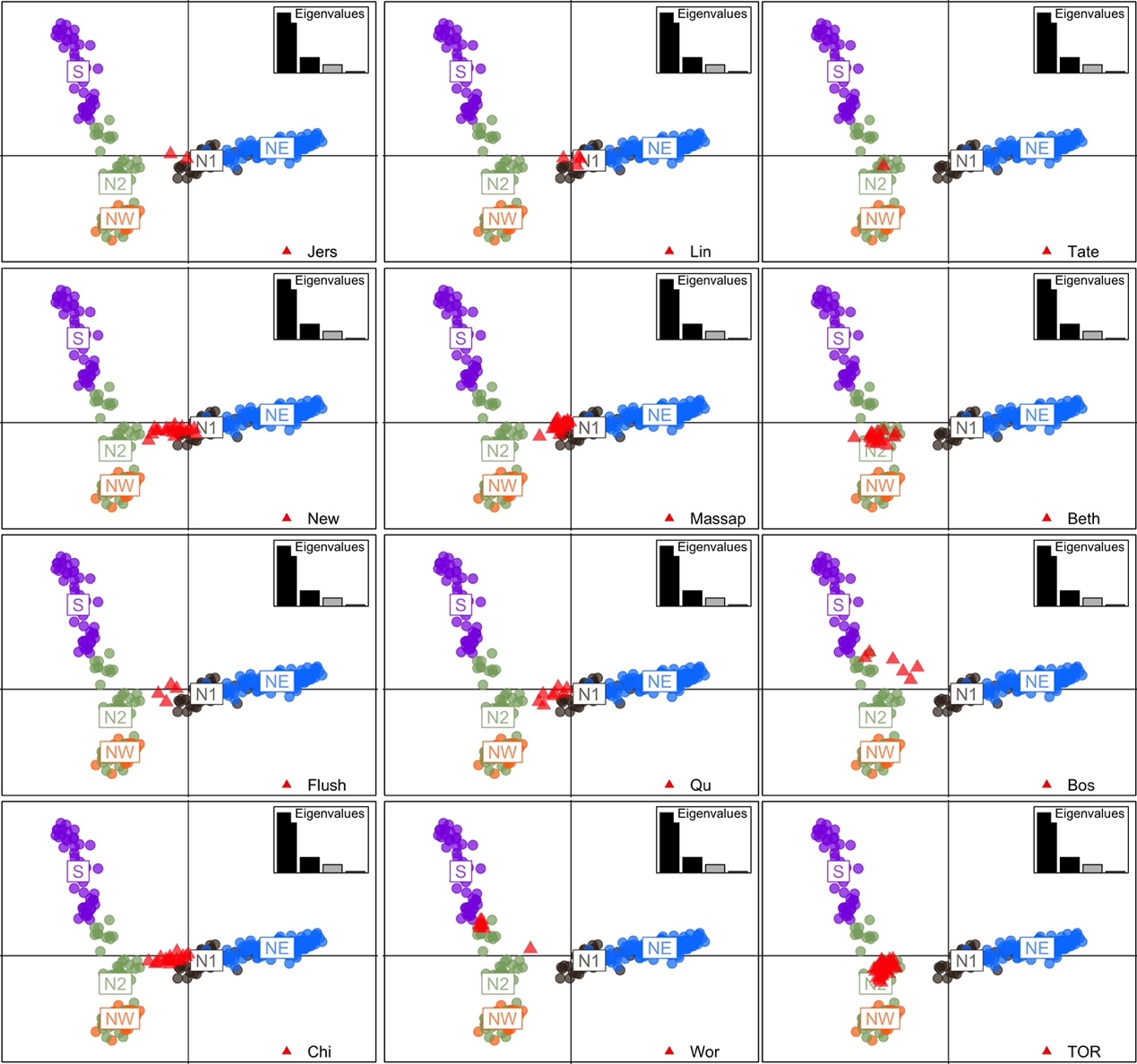
*

**Figure S7:** DAPC assignment plot for each invasive locality (red triangles) relative to five native *A. glabripennis* populations (light-colored circles). Native range: NE - Northeast China; NW - Northwest China; N1 - North China one; N2 - North China two; S - South China. Invasive range: Jers - Jersey, NJ, US; Lin - Linden, NJ, US; Tate - Tate, OH, US; New - New York, NY, US; Massap - Massapequa, NY, US; Beth - Bethel, OH, US; Flush - Flushing, NY, US; Qu - Queens, NY, US; Bos - Boston, MA, US; Chi - Chicago, IL, US; Wor - Worcester, MA, US; TOR - Toronto, Canada.


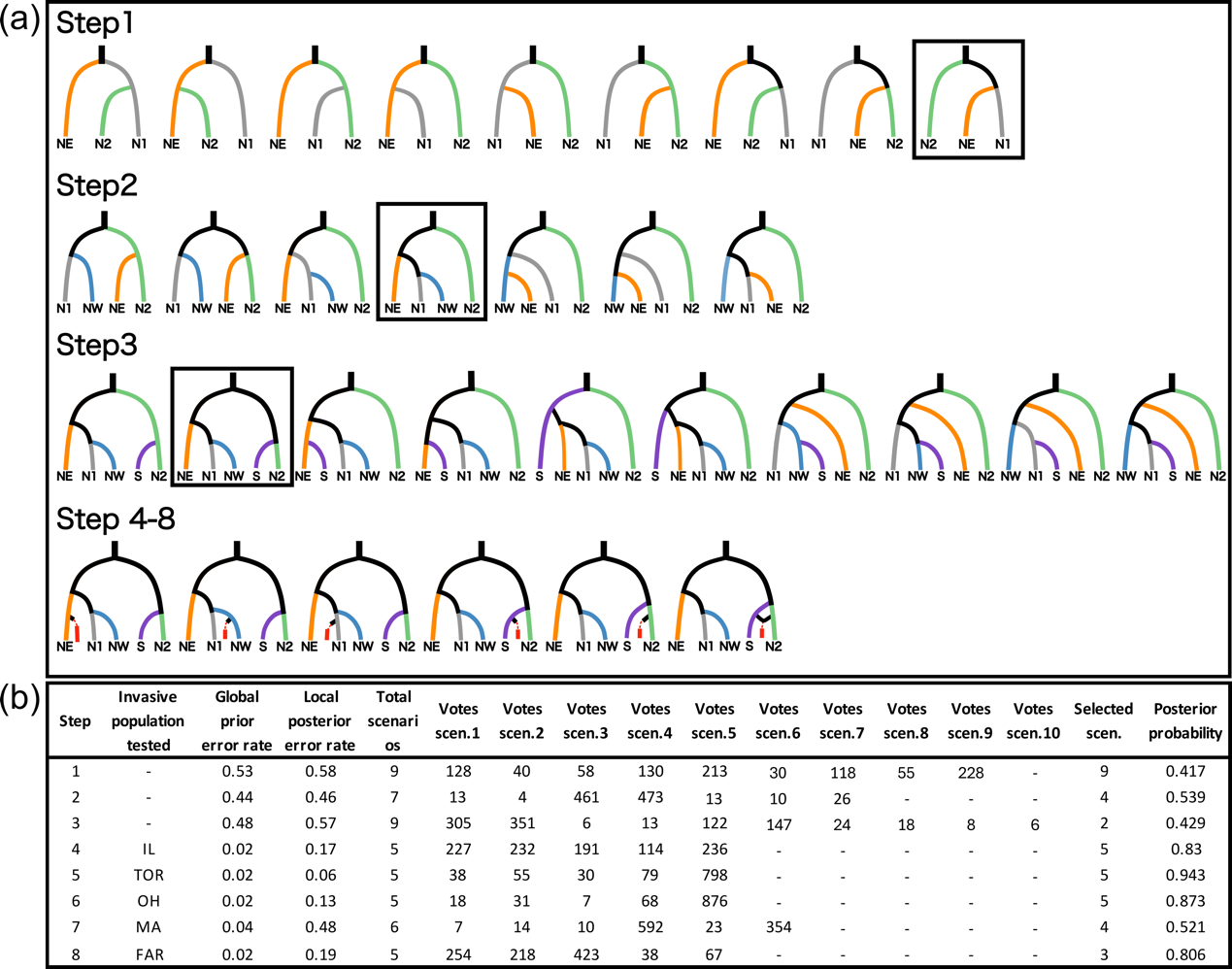


**Figure S8:** (a) Schematic representation of hierarchical relationships among native and invasive *A. glabripennis* populations for stepwise DIYABC model selection and analysis. Native populations are colored as in Figure 4, while unsampled ancestral populations are shown in black, and invasive populations are highlighted in red, with dashed lines indicating bottleneck events. The invasive populations included in the analysis are Illinois (IL), Toronto (TOR), Ohio (OH), Massachusetts (MA), and Farmingdale (Far, New York). In Steps 1 to 3, the scenarios framed in black are the optimal ones with the highest posterior probability. Steps 4 to 8 focus on testing invasive populations individually. Step four is the analysis for the invasive population of IL, testing five scenarios (the first five ones). Step five is the analysis for TOR (five scenarios). Step six is for OH (five scenarios). Step seven is for MA, which includes one extra scenario with a mixed ancestor (the last column) exclusively for Massachusetts (MA). Step eight is for Far (five scenarios). (b) Error rates and votes for best scenario of each step in DIYABC analysis. Prior error rate is calculated by using a set of simulated data from priors and computing the average Type I and Type II errors. Posterior error = 1 - (posterior probability of the best scenario). Posterior probability is estimated by comparing the genetic variation between observed and simulated genotypes with the selected scenario. Votes of Sce. 1-10 indicates the number of trees designated to each scenario per step. PP indicates the posterior probability for the optimal scenario, i.e., the scenario with the most votes of tree.


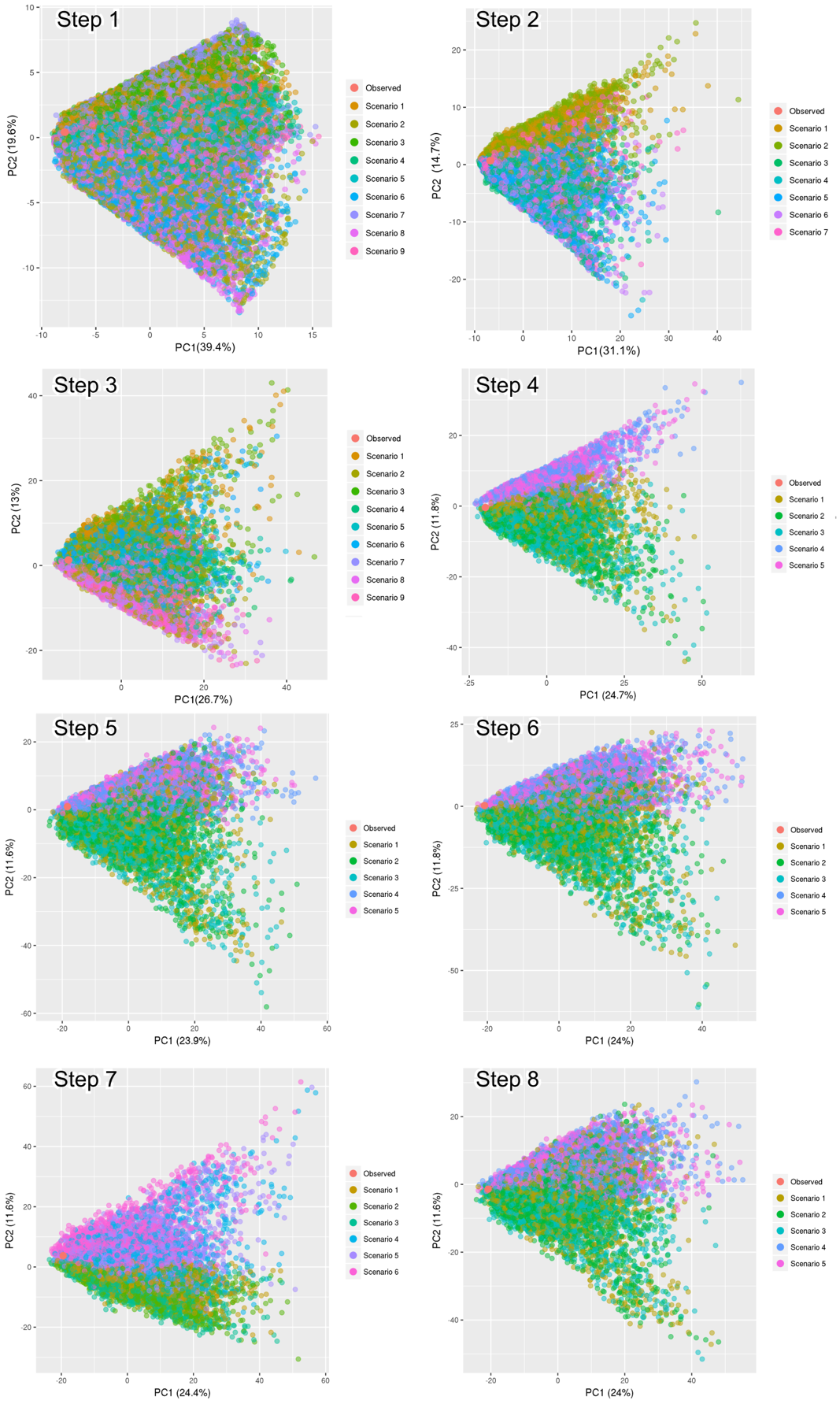


**Figure** **S9:** PCA for model checking for each step in DIYABC. The corresponding scenarios are shown in Figure S8. The PCA uses summary statistics as components of the feature vector to evaluate the selected model.
